# Comparative analysis of fractional thermoelastic vibrations of a nonlocal nanobeam exposed to travelling and static thermal loads

**DOI:** 10.1038/s41598-026-39005-5

**Published:** 2026-02-09

**Authors:** Rakhi Tiwari, Gopal Kumar Gupta, Om Namha Shivay

**Affiliations:** 1 Department of Mathematics, B.R.A. Bihar University, Muzaffarpur, 842001 India; 2 Symbosis Institute of Technology, Nagpur Campus, Symbosis International (Deemed University), Pune, 440008 India; 3 Department of Mathematics, SAS, VIT University, Chennai, 600127 India

**Keywords:** Nano scale beam, Non-locality, Fractional derivative, MGT model, Dynamic load, Ramp type load, Engineering, Materials science, Mathematics and computing, Physics

## Abstract

Present innovative investigation comprises a new approach to analyse the fractional order vibration behaviour of thermo-mechanical waves in a non-localized Nano scale beam affected from a travelling thermal load with constant velocity and ramp type thermal load dependent on time within the framework of nonlocal theory of elasticity. Recently developed Moore-Gibson Thompson (MGT) heat transport model coupled with fractional order thermo-elasticity is utilized to evaluate the analytical results of significant physical fields—temperature, lateral deflection, displacement, cross-sectional elastic moment and thermal stress. Inclusion of the theories of nonlocal elasticity and factional order thermoelasticity in thermal conduction model enables it to capture the size-dependent effects and memory effects in heat conduction at the Nano scale. Laplace transform algorithm is facilitated to determine the closed-form solutions. Depth analysis of graphical results characterizes and analyses the impacts of the important quantities such as fractional order parameter, velocity of the dynamic load, time relaxation quantity and non-local parameter on the field variables. Quantitative results reveal that fractional order quantity and nonlocal effect prominently affect the amplitude, frequency and stability of thermo-elastic vibrations. Significance of MGT heat conduction model is observed by comparing the computational outcomes to the results obtained under previous established heat transfer models – GreenNaghdi- II (GN-II), GreenNaghdi - III (GN-III), Lord and Shulman (LS model) and classical theory (CL). Results determined under MGT model express more finite and stable characteristics of thermo-elastic waves inside the beam compared to the other theories of heat transfer. This study emphasises the significance of applied research in revealing the important properties of Nano scale structures that are observed to be especially advantageous in industry and mechanical engineering.

## Introduction

Nanotechnology expresses the study of materials and structures at the subatomic level to establish new structures that work at the Nano scale. Basically, the term ‘Nanotechnology’ has provided a new direction to the present era by encompassing numerous applications across several industries in terms of computing, analysing, bio processing, sensors, micro/nano electromechanical systems, construction, mechanical and civil engineering^1,2^. Among all applications, Micro/Nano electromechanical systems have attracted the researchers as it refers to an integrated network system of Micro/Nano sensors, actuators and microelectronic devices which are found to be applicable for fabricating many devices like Nano wires, microscope, Nano probes and Nano actuators^3^.

Fourier^4^ derived the law of heat transfer considering the thermo-mechanical coupling effects inside the system that consists of parabolic nature of heat transfer and consequently predicts infinite speed of thermal waves. In this way, this law speculates the results against the realistic nature of waves. Later on, other generalized models of heat transfer such as Lord - Shulman model, Tzou model of two phase lags, Green-Naghdi model of heat conduction etc^5–9^. were discovered where the theory of time relaxation parameters are included. However, the results under the influences of above mentioned heat conduction models were found to be inappropriate especially for the experiments suffering from microstructural effects.

Micro/Nano electromechanical structures (MEMS/NEMS) are composed of thin beam having length of order approx. 10 to 100 microns^10^. Therefore, their size-dependent effects are pronounced and they affect the thermo-coupling mechanism inside the structures^11,12^. Due to this ambiguity, conventional heat transport models exhibit their inability to capture the micro/nano structural effects. For the sake of avoiding these issues, current study has been carried out under the frame of currently established heat transfer theory which is gaining fame as Moore-Gibson-Thomson (MGT) model of thermoelasticity proposed by Quintanillla^13^. Moore-Gibson-Thomson model of heat conduction is remarked as the modification of Lord-Shulman (LS) model and Green-Naghdi- III (GN-III) model of thermoelasticity. Recently, several studies are reported under the effects of Moore Gibson Thomson heat conduction model^14–17^.

In Nano structures which are affected from the temperature dependent surrounding, thermo-mechanical deformation and stress have been become pronounced. This situation may create instability and structural failure. To overcome this issue, scientists and researchers realized that fractional order derivatives are observed to be more compatible for such kinds of systems compared to the conventional integer order derivatives. Derivatives having fractional order capture the memory effect which implies that the current stage of a system depends upon the past stage which reflects the realistic nature of the behaviour of systems. Currently, a popular definition of fractional order derivative was proposed by Caputo^18^ which supports to the accurate modelling of anomalous diffusion processes with better precision. Excellent property of Caputo fractional derivative is observed in its ability to preserve the non-local characteristics that became it an attractive definition of fractional order derivative for modelling of complex systems. Abbas and Hobini^19^ derived the analytical solutions for thermo elastic damping in a Nano scale beam using fractional order derivatives in heat transfer model. Peng et al.^20^ investigated transient thermo-elastic behaviour of nano beam in the framework of fractional heat transfer theory. Abouelregal and Dargail^21^ studied memory effects inside functionally graded Nano beam caused by fluctuating thermal load. Abouelregal and Tiwari^22^ performed computational analysis of thermo elastic vibrations for a functionally graded non local Nano beam affected from an instantaneous thermal load. Very recently Mondal et al.^23^ analysed the dynamics of Nano resonator affected from an oscillating thermal load in two temperature thermo elastic model.

Eringen^24,25^ established nonlocal theory in continuum mechanics which was noticed to be successful for capturing thermo-mechanical interactions for the small-scale structures and materials. Local theory of elasticity speculates that the continuous distributed nature of particles and stress at a point inside the material uniquely depend the strain at the same point while non-local continuum theory has the ability to capture the impact of all positions of the body at a unique physical point. Consequently, nonlocal theory expresses the information on the long range forces for atoms and molecules which permit for the insertion of an internal length scale parameter in the formulation. Zenkour et al. established^26^ a refined nonlocal theory for the thermo elastic vibrations of Nano beam exposed to the ramp-type heat. Arhami et al.^27^ analysed nonlocal thermo elastic Nano-scale beam under Casimir force. Tiwari et al.^28^ studied nonlocal thermo-mechanical waves inside Nano scale resonators. Recently, significant investigations were performed by the researchers that explore the importance of nonlocal and memory effects in context of generalized thermoelasticity^29–37^.

Many significant studies expressing thermo-mechanical vibrations in Nano scale beam have been reported in past. However, those investigations have disregarded the fractional order vibrational behavior of nonlocal Nano beam affected from the combined thermal loadings- dynamic thermal load and ramp type heat dependent on ramp duration under the framework of thermal conduction model with phase lags. This negligence limits to capture the microstructural interactions, nonlocal effects and memory effects during the travelling of thermo-mechanical waves inside the Nano devices which affects the functioning and sensitivity analysis of Nano/Micro structures. For the purpose of filling this gap, current investigation examines the fractional and nonlocal behaviour of thermo-elastic vibrations inside Nano scale beam which is affected from a ramp type thermal load at its boundary and a dynamic thermal load inside the structure. Mathematical modelling is formulated by adopting a recent famous Moore Gibson Thompson (MGT) thermo elastic model which is constructed by the generalization of Lord Shulman (LS) model of thermal conduction and Green-Naghdi model of heat transfer. The newness and novelty of this investigation is based on the inclusion of size-dependent nonlocal effects and memory effects with the more generalized thermal conduction model including relaxation time which enables a more realistic representation of heat transport with finite and stable characteristics. Moreover, the attractiveness of this study is noticed on the comparative assessment of the computational results that provide the right direction to the scientists and researchers to understand the suitability of heat transport model at the time of fabricating and designing of Nano scale devices and structures.

## Establishment of the mathematical model of the problem

**Fig. 1:**
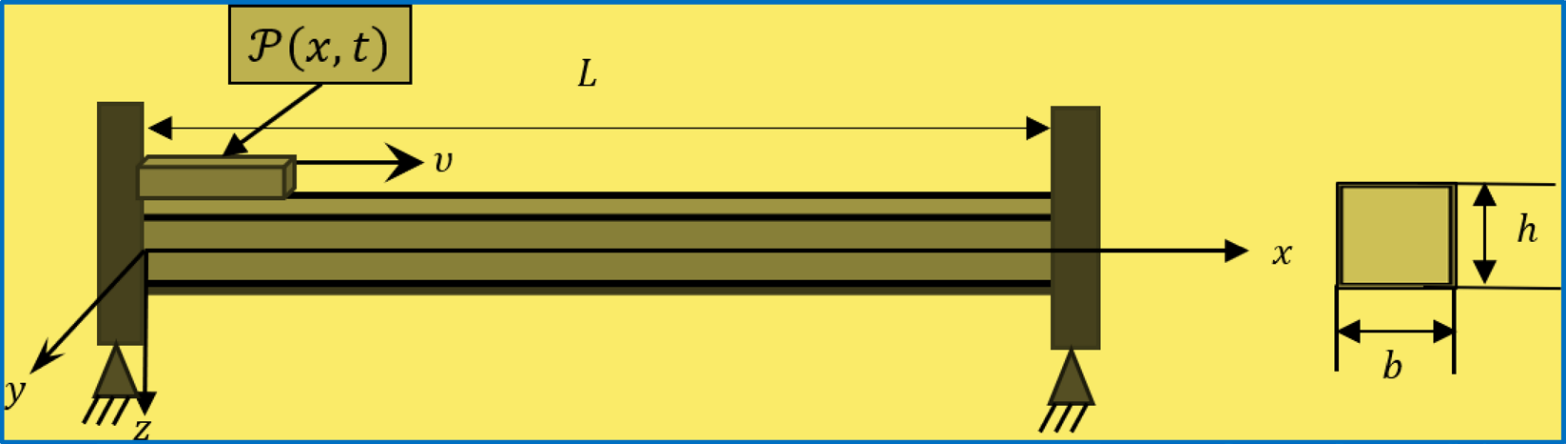
Simply supported Euler-Bernoulli Nano-beam under affected from a dynamic load internally.

Figure 1 outlines the representation of a thin Euler-Bernoulli Nano-beam simply supported on both sides at its boundary. Dimension of the Nano-beam with a symmetric cross-section are considered to be $$\:L,\:b,\:h$$ as its length, breadth and thickness, respectively. Beam suffers from a dynamic thermal load moving along its length having a fixed velocity $$\:v$$; while the outer boundary of beam is exposed to ramp type heating load. Shear deformations are observed to be nominal due to high aspect ratio (𝐿/ℎ), and can be neglected during the study. Consequently, thin Nano-beam consideration permits the concept of Euler-Bernoulli beam theory. At the starting period, Nano-beam is assumed to be free from stress and strain and is kept at a uniform reference temperature: $$Kindly remove this line.$$

In the absence of external heat source, the generalized heat conduction equation in context of Caputo fractional MGT theory without heat source (*Q* = 0) is given by^13^

1 $$\:{K\dot{\theta\:}}_{,ii}+{K}^{*}{\theta\:}_{,ii}=\left(\:1+\frac{{\tau\:}_{q}^{\alpha\:}{D}^{\alpha\:}\:}{{\Gamma\:}\left({\upalpha\:}+1\right)}\right)\left[\rho\:{c}_{E}\ddot{\theta\:}-z\gamma\:{T}_{0}\:\frac{{\partial\:}^{4}\:\ddot{w}}{\partial\:{x}^{2}\partial\:{t}^{2}}\right].$$

Here, fractional derivatives of order $\:\alpha\:$ lies between 0 and 1 (0 < $\:\alpha\:$ ≤ 1).

Special cases:

1. $\:\alpha\:=1$ converts Eq. (1) into generalized Moore Gibson Thompson heat conduction equation with conventional derivatives.
2. $\:\alpha\:=1,{K}^{*}=0\:$corresponds to the Lord Shulman model (LS) of generalized thermoelasticity. 
3. $\:{K}^{*}=0,\:{\tau\:}_{q}=0$ exhibits the classical thermoelastic model (CL) of thermoelasticity.
4. Keeping $\:{\tau\:}_{q}=0$ into Eq. (1), Green-Naghdi-III (GN-III) model of heat transfer is obtained.
5. $\:K=0,\:{K}^{*}>0$ corresponds to Green- Naghdi-II (GN-II) theory of thermoelasticity.

Components of displacement field $\:u$ and dilation $\:e$ are expressed as:

2 $$\:u=\:-\:z{w}_{x},\mathrm{v}=\:0,w\left(x,\:y,\:z,\:t\right)=\:w\left(x,\:t\right)e={u}_{x}=-z\:{w}_{xx}.\:$$

Relation between non-local stress, strain and temperature fields are defined as^38^:

3 $$\:{\sigma\:}_{xx}-\xi\:\frac{{\partial\:}^{2}{\sigma\:}_{xx}}{\partial\:{x}^{2}}=\:-Ez{w}_{xx}-{\alpha\:}_{t}E\theta\:$$

where $$\:\gamma\:=\frac{E\:{\alpha\:}_{t}}{(1-2\upsilon\:)}$$.

Equation of motion exhibiting the vibration of nano-beam is written as:

4 $$\:\frac{{\partial\:}^{2}M}{\partial\:{x}^{2}}=\rho\:A\frac{{\partial\:}^{2}w}{\partial\:{t}^{2}}\:,\:\:$$

$$\:\mathrm{w}\mathrm{h}\mathrm{e}\mathrm{r}\mathrm{e}\:A=bh$$

$\:M\left(x,t\right),$ is recognized as the flexural moment and mathematically, it is presented as:

5 $$\:M=\:{\int\:}_{-\frac{h}{2}}^{\frac{h}{2}}\left(z\sigma\:\right)dz.$$

Since Eringen nonlocal effect is applicable for the Nano scale only. Therefore, incorporating the concept of non-local effect, relation between moment and lateral deflection becomes

6 $$\:M-\:\xi\:\frac{{\partial\:}^{2}M}{\partial\:{x}^{2}}=\:-E\:{I}_{0}\frac{{\partial\:}^{2}w}{\partial\:{x}^{2}}-{\alpha\:}_{t}E{I}_{0}{M}_{T}.$$

The parameter 𝜉 = (𝑒_0_𝑎)^2^, represents the nonlocal quantity which is constituted material constant 𝑒_0_ and internal characteristic length 𝑎. This parameter works for size- dependent impact. As its limitation, it is especially applied for the structures at Nano scale. That’s why it is highly applicable for thin Nano beam known as Euler Bernoulli Nano beam.

Thermal moment $\:{M}_{T}$ is given by^38,39^

7 $$\:{M}_{T}=\:\frac{12}{{h}^{3}}{\int\:}_{-\frac{h}{2}}^{\frac{h}{2}}\left(z\theta\:\right)dz.$$

Since, Nano beam is affected from a dynamical thermal load. Therefore, equation for motion for Nano beam is given by

8 $$\:\frac{{\partial\:}^{2}M}{\partial\:{x}^{2}}+P\left(x,t\right)=\rho\:A\frac{{\partial\:}^{2}w}{\partial\:{t}^{2}}.\:\:$$

Substituting the relation of moment from Eq. (8) to Eq. (6), we obtain

9 $$\:\:\:\:\:\:\:\:\:\:\:\:\:\:\:\:\:\:\:\:\:\:\:\:\:\:\:\:\:\:M=\:\rho\:A\xi\:\frac{{\partial\:}^{2}w}{\partial\:{t}^{2}}-E{I}_{0}\frac{{\partial\:}^{2}w}{\partial\:{x}^{2}}-{\alpha\:}_{T}E{I}_{0}{M}_{T}-\xi\:P\left(x,t\right)$$

Finally, the equation of motion for the Euler Nano beam including nonlocal elasticity is expressed as:

10 $$\:E{I}_{0}\frac{{\partial\:}^{4}w}{{\partial\:x}^{4}}+\rho\:A\:\frac{{\partial\:}^{2}}{{\partial\:t}^{2}}\left[1-\xi\:\frac{{\partial\:}^{2}}{{\partial\:x}^{2}}\right]w-\left(\:1-\xi\:\frac{{\partial\:}^{2}}{{\partial\:x}^{2}}\right)P=\:-{\alpha\:}_{T}E{I}_{0}\frac{{\partial\:}^{2}{M}_{T}}{\partial\:{x}^{2}}.$$

Here, $$\:{I}_{0}=\frac{b{h}^{3}}{12}$$.

After arranging the terms of Eq. (10), it yields

11 $$\:{D}^{4}w+\:\frac{\:\rho\:A}{E{I}_{0}}\left(1-\xi\:{D}^{2}\right)\ddot{w}-\frac{\:1}{E{I}_{0}}\left(1-\xi\:{D}^{2}\right)P=-{\alpha\:}_{t}{D}^{2}{M}_{T}.$$

$\:D$ reflects the derivative w.r.t space variable $\:x.$

Heat transfer equation (Eq. (1)) can be expressed as:

12 $$[{K}^{*}+K\frac{\partial\:}{\partial\:t}]{D}^{2}\theta\:=(\:1+\:\frac{{\tau\:}_{q}^{\alpha\:}\:{D}^{\alpha\:}}{{\Gamma\:}\left(\alpha\:+1\right)}\left)\right(\rho\:{c}_{e}\ddot{\theta\:}-\:z\gamma\:{T}_{0}{D}^{2}\ddot{w)}.$$

Equation (9) is recognized as:

13 $$\:\:M=\:\rho\:A\xi\:\ddot{w}-E{I}_{0}{D}^{2}w-{\alpha\:}_{T}E{I}_{0}{M}_{T}-\xi\:P.$$

## Closed-form solutions along the direction of thickness of beam

It is assumed as the temperature alterations along the thickness of the beam follow sinusoidal behaviour. Hence, temperature distributions are presented as:

14 $$\:\:\theta\:\left(x,p,z,t\right)=\:\psi\:\left(x,t\right)\mathrm{sin}\left(pz\right),$$

where $$\:p=\frac{\pi\:}{h}$$.

$\:\psi\:\left(x,t\right)$ denotes the temperature amplitude function and $\:\mathrm{sin}\left(pz\right)\:$ yields the oslillatory distribution of temperature field along the thickness $\:h$ with the condition that temperature gradient achieves zero numerical value at the boundaries *z = ± h/2*.

Introducing Eq. (14) into Eqs. (12)-(14), we achieve

15 $$\:{D}^{4}w+\:\frac{\:\rho\:A}{E{I}_{0}}\left(1-\xi\:{D}^{2}\right)\ddot{w}+\:\frac{24\:{\alpha\:}_{T}}{{\pi\:}^{2}{h}^{3}}\:{D}^{2}\psi\:=\:\frac{\:1}{E{I}_{0}}\left(1-\xi\:{D}^{2}\right)P.$$

16 $$[{K}^{*}+K\frac{\partial\:}{\partial\:t}]{(D}^{2}-{p}^{2})\psi\:=(\:1+\:\frac{{\tau\:}_{q}^{\alpha\:}\:{D}^{\alpha\:}}{{\Gamma\:}\left(\alpha\:+1\right)})(\rho\:{c}_{e}\ddot{\psi\:}-(\:{p}^{2}{h}^{3}\gamma\:{T}_{0}/24\left){D}^{2}\ddot{w}\right).$$

17 $$\:M=\:\rho\:A\xi\:\ddot{w}-E{I}_{0}{D}^{2}w-\frac{24\:{\alpha\:}_{T}}{{\pi\:}^{2}h}{\alpha\:}_{T}E{I}_{0}{M}_{T}-\xi\:P.$$

A concentrated external moving load $\:P(x,t)$ with constant strength $\:\:{Q}_{0}$ is traveling along the axis of the Nano-beam at a constant speed. Mathematically, it can be mentioned as:

18 $$\:P={Q}_{0}\delta\:\left(x-vt\right)$$.

For the purpose of simplifying the governing Eqs. (15)-(17), we introduce the following dimensionless terms:

$$\:\left({x}^{{\prime\:}},{u}^{{\prime\:}},{w}^{{\prime\:}},{z}^{{\prime\:}}\right)=\:{c}_{0}n\left(x,u,w,z\right);$$

$$\:\left({t}^{{\prime\:}},{\tau\:}_{q}^{{\prime\:}},{\tau\:}_{\theta\:}^{{\prime\:}}\right)={c}_{0}^{2}n\left(t,{\tau\:}_{q}\right);$$

$$\:{\xi\:}^{{\prime\:}}=\:{n}^{2}{c}_{0}^{2}\xi\:,\:{\:\:\psi\:}^{{\prime\:}}=\frac{\psi\:}{{T}_{0}},\:\:{P}^{{\prime\:}}=\:\frac{A}{E{I}_{0}}P,\:{m}^{{\prime\:}}=\:\frac{1}{n{c}_{0}E{I}_{0}},\:\:{c}_{0}=\:\sqrt{\frac{E}{\rho\:}}\:,\:n=\:\frac{\rho\:{c}_{0}}{K}.$$

Equations (15–17) are converted into the following dimensionless form:

19 $$\:{D}^{4}\omega\:+\:\frac{12}{{h}^{2}}\left(1-\xi\:{D}^{2}\right)\ddot{w}+\:\left(\frac{24\:{\alpha\:}_{T}{T}_{0}}{{\pi\:}^{2}h}\right)\:\frac{{\partial\:}^{2}\psi\:}{{\partial\:x}^{2}}=\:\left(P-\xi\:\frac{{\partial\:}^{2}P}{{\partial\:x}^{2}}\right).$$

20 $$\:M=\left(\frac{12\xi\:}{{h}^{2}}\right)\ddot{w}-\:{w}_{xx}-\frac{24\:{\alpha\:}_{T}}{{\pi\:}^{2}h}\psi\:-\xi\:P.\:\:\:\:\:\:\:\:\:\:\:\:\:\:\:\:\:\:\:\:\:\:\:\:\:\:\:\:\:\:\:\:\:\:\:\:\:\:\:\:\:\:\:\:\:\:\:\:\:\:\:\:\:\:\:\:\:\:\:\:\:\:\:\:\:\:\:\:\:\:\:\:\:\:\:\:\:\:\:\:\:\:\:\:\:\:\:\:\:\:\:\:\:\:\:$$

21 $$\:\left(\left(\frac{{K}^{*}}{{{c}_{0}n}^{2}K}\right)\:+\frac{\partial\:}{\partial\:t}\right)\:\:{(D}^{2}-{p}^{2})\psi\:=\left(1+\:\frac{{\tau\:}_{q}^{\alpha\:}\:{D}^{\alpha\:}}{{\Gamma\:}\left(\alpha\:+1\right)}\right)\left[\ddot{\psi\:}-\left(\frac{{\pi\:}^{2}h\gamma\:}{24\:Kn}\right){D}^{2}\ddot{w}\right].$$

Initial conditions in dimensionless form are assumed as:

22 $$\:w\left(x,0\right)=\dot{w}\left(x,0\right)=0,\:\psi\:\left(x,0\right)=\dot{\psi\:}\left(x,0\right)=0.$$

In the case of simply supported Nano-beam, following boundary conditions at both ends of beam are taken into account-

23 $$\:w\left(x,t\right)=\frac{{\partial\:}^{2}w(x,t)}{\partial\:{x}^{2}}=0\:\mathrm{a}\mathrm{t}\:x=0,L.$$

Additionally, nano-beam is considered to be thermally loaded with ramp type heat at its first boundary. Therefore

24 $$\:\theta\:\left(0,t\right)={\psi\:}_{0}f(0,t)\:\mathrm{w}\mathrm{h}\mathrm{e}\mathrm{r}\mathrm{e}\:f\left(0,t\right)=\left\{\begin{array}{c}0,\:\:\:\:\:\:\:\:\:\:\:\:t\le\:0\\\:\frac{t}{{t}_{0}},\:\:\:\:\:0\le\:t\le\:{t}_{0}\\\:1,\:\:\:\:\:\:\:t>{t}_{0}\end{array}.\right.$$

25 $$\:\frac{\partial\:\theta\:(L,t)}{\partial\:x}=0.$$

## Analytical solutions in transformed domain using Laplace transform methodology

Adopting the methodology of Laplace Transform in coupled Eqs. (19–21), we obtain.

26 $$[{D}^{4}\stackrel{-}{w}-{a}_{1}\xi\:{D}^{2}\stackrel{-}{w}-{a}_{1}\stackrel{-}{w}]=\:{-a}_{2}{D}^{2}\stackrel{-}{\psi\:}+g(s)\:{e}^{-\left(\frac{s}{v}\right)x}$$

27 $$\:\stackrel{-}{M}=\:{-(D}^{2}{-a}_{1}\xi\:)\stackrel{-}{w}-{a}_{2}\stackrel{-}{\psi\:}-\xi\:\left(\frac{{Q}_{0}{e}^{-\left(\frac{s}{v}\right)x}}{v}\right)$$

28 $$\:{(D}^{2}-{p}^{2}-{a}_{4})\stackrel{-}{\psi\:}=-{a}_{3}{{a}_{4}D}^{2}\stackrel{-}{w}$$

$$\:\mathrm{w}\mathrm{h}\mathrm{e}\mathrm{r}\mathrm{e}\:\:{a}_{1}=\:\frac{12\:{s}^{2}}{{h}^{2}}\:,\:\:\:{a}_{2}=\left(\frac{24\:{\alpha\:}_{T}{T}_{0}{D}^{2}\psi\:}{{\pi\:}^{2}h}\right),\:{a}_{3}=\left(\frac{{\pi\:}^{2}h\gamma\:}{24\:kn}\right),\:\:{a}_{4}=\frac{\left(\:1+\:{\tau\:}_{q}^{\alpha\:}\:{s}^{\alpha\:}\right){s}^{2}}{{(K}^{*}/{{c}_{0}n}^{2}K)\:+s)}\:,\:\:g\left(s\right)=\frac{{Q}_{0}}{v}\left(1-\xi\:\left(\frac{{s}^{2}}{{v}^{2}}\right)\right).$$

Solving coupled Eqs. (26) and (28) in terms of lateral deflection $\:\stackrel{-}{w}$, following six order differential equation in terms of $\:\stackrel{-}{w}$ is derived-

29 $$\:{[D}^{6}-A{D}^{4}+B{D}^{2}-C]\stackrel{-}{w}=g(s\left)\right[\frac{\:{s}^{2}}{{v}^{2}}-{p}^{2}-{a}_{4}]{e}^{-\left(\frac{s}{v}\right)x}.$$

Here, the constants are determined as:

$$\:A=\left[\left({a}_{1}\xi\:+{p}^{2}+{a}_{4}\right){a}_{2}{a}_{3}{a}_{4}\right],\:B=[{a}_{1}+{a}_{1}\xi\:\left({p}^{2}+{a}_{4}\right)],\:{C=a}_{1}\left({p}^{2}+{a}_{4}\right).$$

On solving Eq. (29), analytical solution for the lateral deflection $\:\stackrel{-}{w}$ in transformed domain is determined as:

30 $$\:\stackrel{-}{w}=\sum\:_{j=1}^{3}({m}_{i}{e}^{-{k}_{i}x}+{m}_{i+3}{e}^{{k}_{i}x})+{m}_{7}\:{e}^{-\left(\frac{s}{v}\right)x}.$$

$\:{{k}_{i}}^{2},\:i=\mathrm{1,2},3,$ reflect the roots of the characteristic equation of Eq. (29).

$$\:{m}_{7}=\frac{g\left(s\right)[\:\frac{\:{s}^{2}}{{v}^{2}}-\:{p}^{2}-{a}_{4}]\:}{{\left(\frac{\:{s}^{2}}{{v}^{2}}\right)}^{6}-A\:{\left(\frac{\:{s}^{2}}{{v}^{2}}\right)}^{4}+B{\left(\frac{\:{s}^{2}}{{v}^{2}}\right)}^{2}-C}.$$

Adopting the similar pattern and using Eqs. (26) and (28), six degree differential equation in terms of temperature $\:\stackrel{-}{\psi\:}$ is evaluated as

31 $$\:{[D}^{6}-A{D}^{4}+B{D}^{2}-C]\:\stackrel{-}{\psi\:}\:=-{a}_{3}{a}_{4}\:g(s)\frac{\:{s}^{2}}{{v}^{2}}{e}^{-\left(\frac{s}{v}\right)x}.$$

Solution of Eq. (31) predicts the analytical solution of temperature field in transformed domain which is expressed as:

32 $$\:\stackrel{-}{\psi\:}=\sum\:_{j=1}^{3}({n}_{i}{e}^{-{k}_{i}x}+{n}_{i}{e}^{{k}_{i}x})+{m}_{8}{e}^{-\left(\frac{s}{v}\right)x}$$.

$$\:{m}_{8}=\:-\frac{{a}_{3}{a}_{4}\:g\left(s\right)\frac{\:{s}^{2}}{{v}^{2}}}{{\left(\frac{\:{s}^{2}}{{v}^{2}}\right)}^{6}-A\:{\left(\frac{\:{s}^{2}}{{v}^{2}}\right)}^{4}+B{\left(\frac{\:{s}^{2}}{{v}^{2}}\right)}^{2}-\:C}\:.$$

In order to obtain $\:{n}_{i}$, Eq. (28) is used in the following way:

33 $$\:{({k}_{i}}^{2}-{p}^{2}-{a}_{4}){n}_{i}=-{a}_{3}{a}_{4}{{k}_{i}}^{2}{m}_{i}\:,$$

which implies$$\:\:{n}_{i}=-\frac{-{a}_{3}{a}_{4}{{k}_{i}}^{2}{m}_{i}}{{({k}_{i}}^{2}-{p}^{2}-{a}_{4})}={q}_{i}{m}_{i}$$.

Equation (32) becomes

34 $$\:\stackrel{-}{\psi\:}=\sum\:_{j=1}^{3}{q}_{i}({m}_{i}{e}^{-{k}_{i}x}+{m}_{i+3}{e}^{{k}_{i}x})+{m}_{8}\:{e}^{-\left(\frac{s}{v}\right)x}$$

Using Eqs. (30) and (34) into Eq. (27), the general solution for the moment $\:\stackrel{-}{M}$ is predicted as:

35 $$\:\stackrel{-}{M}=-\:\sum\:_{j=1}^{3}{({k}_{i}}^{2}-{a}_{i}\xi\:-{a}_{2}{q}_{i}\left)\right({m}_{i}{e}^{{-k}_{i}x}+{m}_{i}{e}^{{k}_{i}x})-\left[{m}_{7}\:\left[\:\frac{\:{s}^{2}}{{v}^{2}}-\:{a}_{1}\xi\:\right]+{a}_{2}{m}_{8}+\frac{\xi\:{Q}_{0}}{v}\right]\:{e}^{-\left(\frac{s}{v}\right)x}.$$

Further, it is arranged as:

36 $$\:\stackrel{-}{M}=\:\sum\:_{j=1}^{3}{r}_{i}({m}_{i}{e}^{{-k}_{i}x}+{m}_{i}{e}^{{k}_{i}x})-{m}_{9}\:{e}^{-\left(\frac{s}{v}\right)x}.$$

$$\:{r}_{i}=-{({k}_{i}}^{2}-{a}_{i}\xi\:-{a}_{2}{q}_{i}),\:{m}_{9}=\left[{m}_{7}\:\left[\:\frac{\:{s}^{2}}{{v}^{2}}-\:{a}_{1}\xi\:\right]+{a}_{2}{m}_{8}+\frac{\xi\:{Q}_{0}}{v}\right].$$

Displacement field is determined as:

37 $$\:u=-z{w}_{x}=z\:\sum\:_{j=1}^{3}({k}_{i}{m}_{i}{e}^{{-k}_{i}x}+{m}_{i+3}{e}^{{-k}_{i}x})+z\:{m}_{7}\frac{s}{v}{e}^{-\left(\frac{s}{v}\right)x}$$

The boundary conditions (Eqs. (22), (23)) are obtained in the Laplace transform domain as:

38 $$\:\stackrel{-}{w}\left(x,s\right)=\frac{{\partial\:}^{2}\stackrel{-}{w}(x,s)}{\partial\:{x}^{2}}=0\:\mathrm{a}\mathrm{t}\:x=0,L;\:\stackrel{-}{\psi\:}\left(0,s\right)={\psi\:}_{0}\left(\frac{1-{e}^{-{t}_{0}s}}{{t}_{0}s}\right)=G\left(s\right).$$

Substituting the boundary conditions in the closed form solutions of physical fields $\:\stackrel{-}{w}$ and $\:\stackrel{-}{\psi\:}$, we find.

For the outer boundary of Nano-beam ($\:x=0)$, following expressions are used

39 $$\:\:\sum\:_{j=1}^{3}({m}_{i}+{m}_{i+3})=-{m}_{7}.$$

40 $$\:\:\sum\:_{j=1}^{3}{{k}_{i}}^{2}({m}_{i}+{m}_{i+3})=-{m}_{7}\frac{\:{s}^{2}}{{v}^{2}}.$$

41 $$\:\:\sum\:_{j=1}^{3}{q}_{i}({m}_{i}+{m}_{i+3})=G\left(s\right)+{m}_{8}$$

For the second boundary of beam ($\:x=L$), we get

42 $$\:\:\sum\:_{j=1}^{3}({m}_{i}{e}^{{-k}_{i}L}+{m}_{i+3}{e}^{{-k}_{i}L})={-m}_{7}{e}^{-\left(\frac{s}{v}\right)L}$$

43 $$\:\:\sum\:_{j=1}^{3}{{k}_{i}}^{2}({m}_{i}{e}^{{-k}_{i}L}+{m}_{i+3}{e}^{{-k}_{i}L})=-{m}_{7}\frac{\:{s}^{2}}{{v}^{2}}{e}^{-\left(\frac{s}{v}\right)L}.$$

44 $$\:\:\sum\:_{j=1}^{3}{k}_{i}{q}_{i}({m}_{i}{e}^{{-k}_{i}L}+{m}_{i+3}{e}^{{-k}_{i}L})={m}_{8}\frac{s}{v}{e}^{-\left(\frac{s}{v}\right)L}.$$

On solving above mentioned equations, we can easily find the values of unknown constants $\:{m}_{i}$ for $\:i=\mathrm{1,2},\mathrm{3,4},\mathrm{5,6}.$

## Numerical results

Current section addresses the computational study of Nano beam of silicon material. Significance of important physical parameters such as fractional parameter $\:\alpha\:$, moving thermal heat source velocity (v), the nonlocal parameter (ξ), and time relaxation parameter $\:{t}_{0}$ are focused on physical fields- lateral deflection, temperature, displacement and thermal moment. It is examined how these parameters influence the distributions of physical fields when the thermo-mechanical waves originate inside the beam. Apart from this, importance of the Moore Gibson Thompson heat transfer model is demonstrated by comparing the numerical results obtained under old classical heat transfer models like GN-II, GN-III, LS model and classical heat transport model.

Quantitative results are derived in physical domain for which it is necessary to convert the analytical solutions from Laplace transform/frequency domain physical/time domain which is a strenuous task. Therefore, for solving this issue, analytical results are numerical inverted into the time domain. The following algorithm is opted to invert the results from Laplace transform domain to the physical domain^40^:

45 $$\:F\left(x,t\right)=\frac{{e}^{pt}}{t}\left(\frac{1}{2}Re\left[\stackrel{-}{F}\left(x,p\right)\right]+Re\sum\:_{n=0}^{N}{\left(-1\right)}^{n}\stackrel{-}{F}\left(x,p+\frac{\mathcal{i}n\pi\:}{t}\right)\right)$$

Here, $\:Re$ stipulates the real part of a complex number, and $\:\mathcal{i}$ denotes the imaginary unit. For the purpose of increasing the speed of convergence of the inversion process, $\:p$ is considered as: $\:p=\frac{4.7}{t}$^41^.

Methodology expressed above becomes an efficacious transformation from the Laplace transform domain to the physical domain.

Numerical values of material parameters are provided in the following Table 1^31,32,34^.

**Table 1:** Quantitative values of material parameters.

Material parameter	Numerical value
Coefficient of thermal expansion *αt*	$\:2.59\times\:{10}^{-6}{K}^{-1}$
Thermal conductivity *K*	156 W/(m K)
Poisson ratio *v*	0.22
Reference temperature $\:{T}_{0}$	$\:293\:K$
Young’s modulus *E*	169GPa
Specific heat $\:{c}_{E}$	$\:713\frac{\mathrm{J}}{\mathrm{k}\mathrm{g}\cdot\:\mathrm{K}}$
Dimensionless time $\:t$	0.25
Material density *ρ*	2330$\:kg{m}^{-3}$
Aspect ratio *L*/ℎ	15
Width-to-thickness ratio *b*/ℎ	0.5
Dimensionless length of beam *L*	1
phase lag $\:{\tau\:}_{q}$	0.15
Evaluation point *z*	ℎ/3

## Detailed technical analysis of computational results

This section explores the effects of important parameters – nonlocal quantity, fractional parameter, time quantity, and the velocity of moving thermal source. Additionally, current section derives the utility of MGT heat transport model compared to the old established models- Green-Naghdi-II, III models, Lord-Shulman model (LS model) and classical model (CL model) of heat transport on the variations of physical fields-temperature, thermal moment, displacement and lateral deflection. In order to explain the influences of each parameter on the field quantities, current section is divided into subsections and each section is devoted to express the impacts of one parameter on the graphical outcomes.

### Influences of various heat transport theories on the behaviour of physical fields

**Fig. 2:**
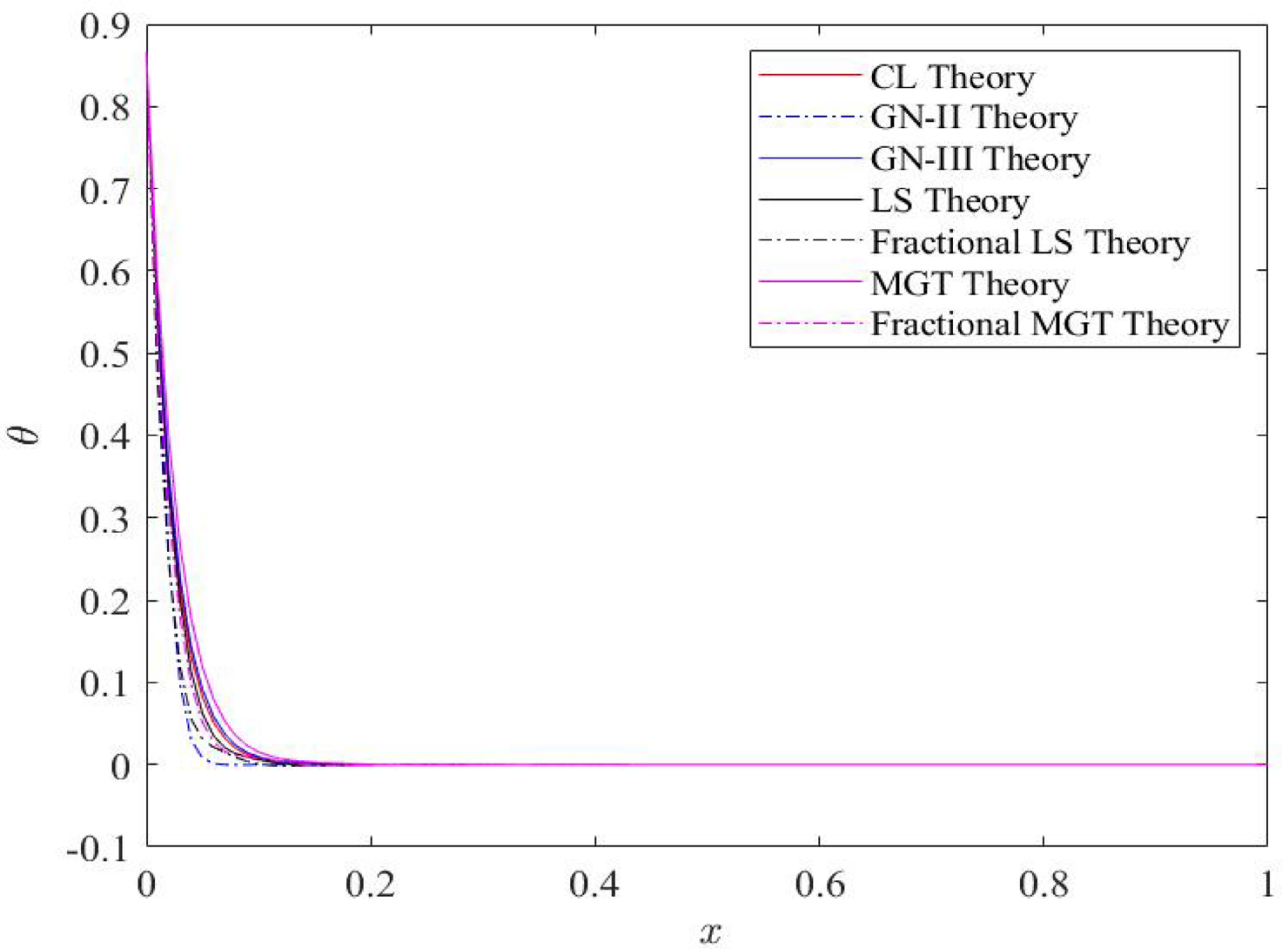
Distributions of temperature field $\:\theta\:$ for distinct heat transfer models.

**Fig. 3:**
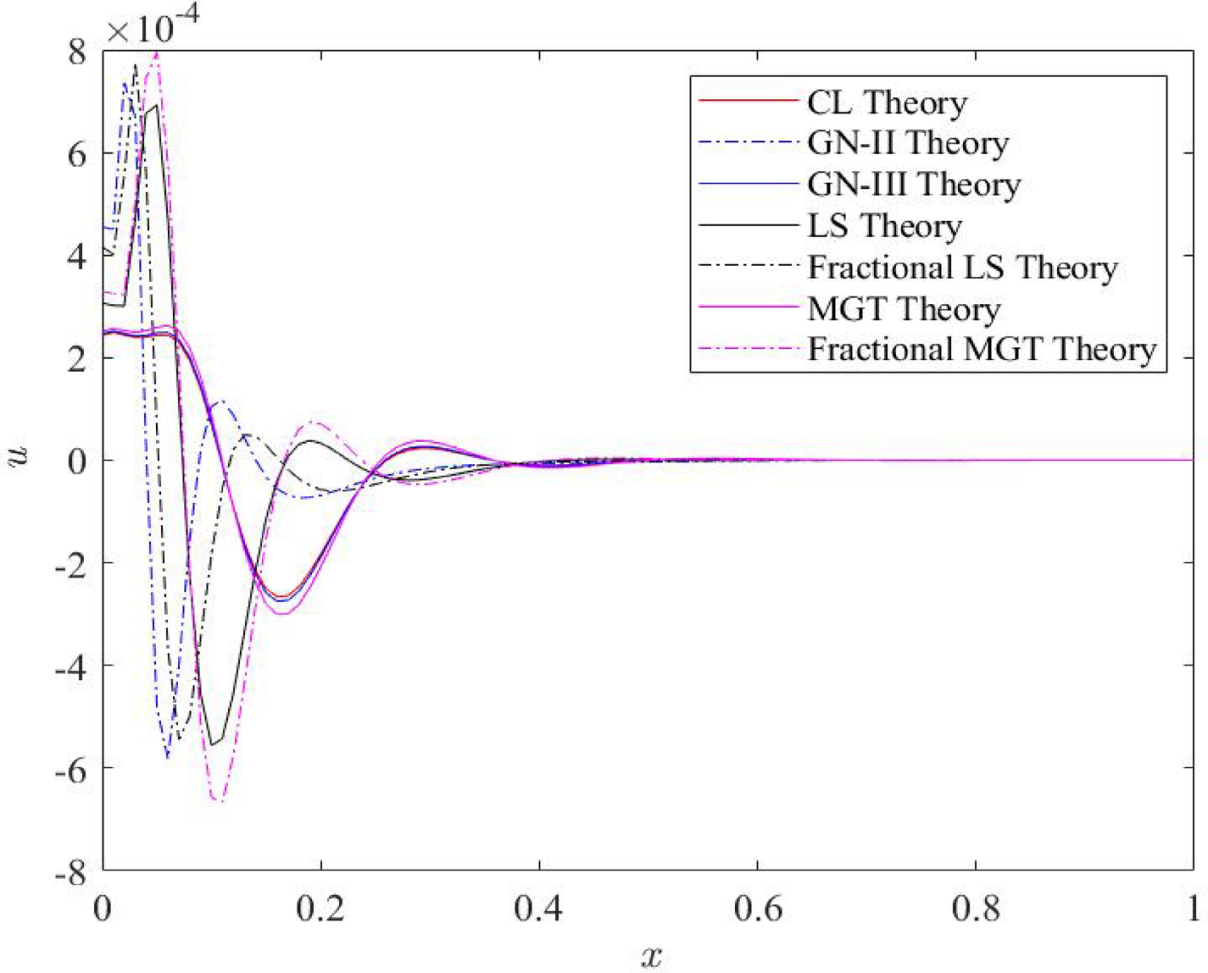
Distributions of displacement field $\:u\:$for distinct heat transfer models.

**Fig. 4:**
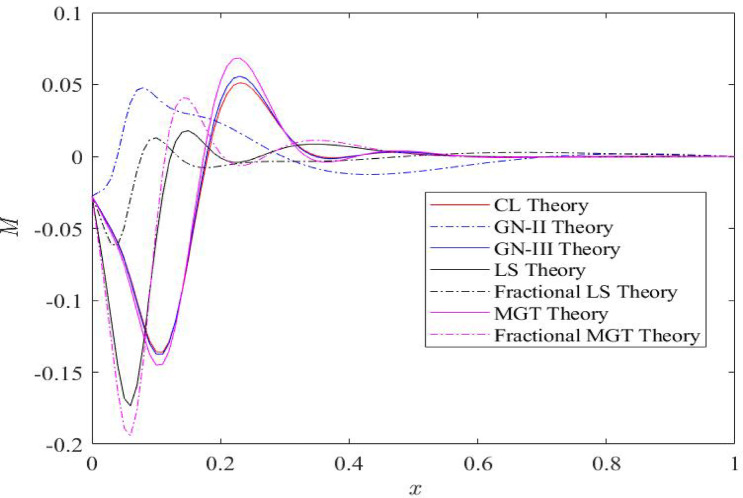
Distributions of thermal moment field $\:M$ for distinct heat transfer models.

**Fig. 5:**
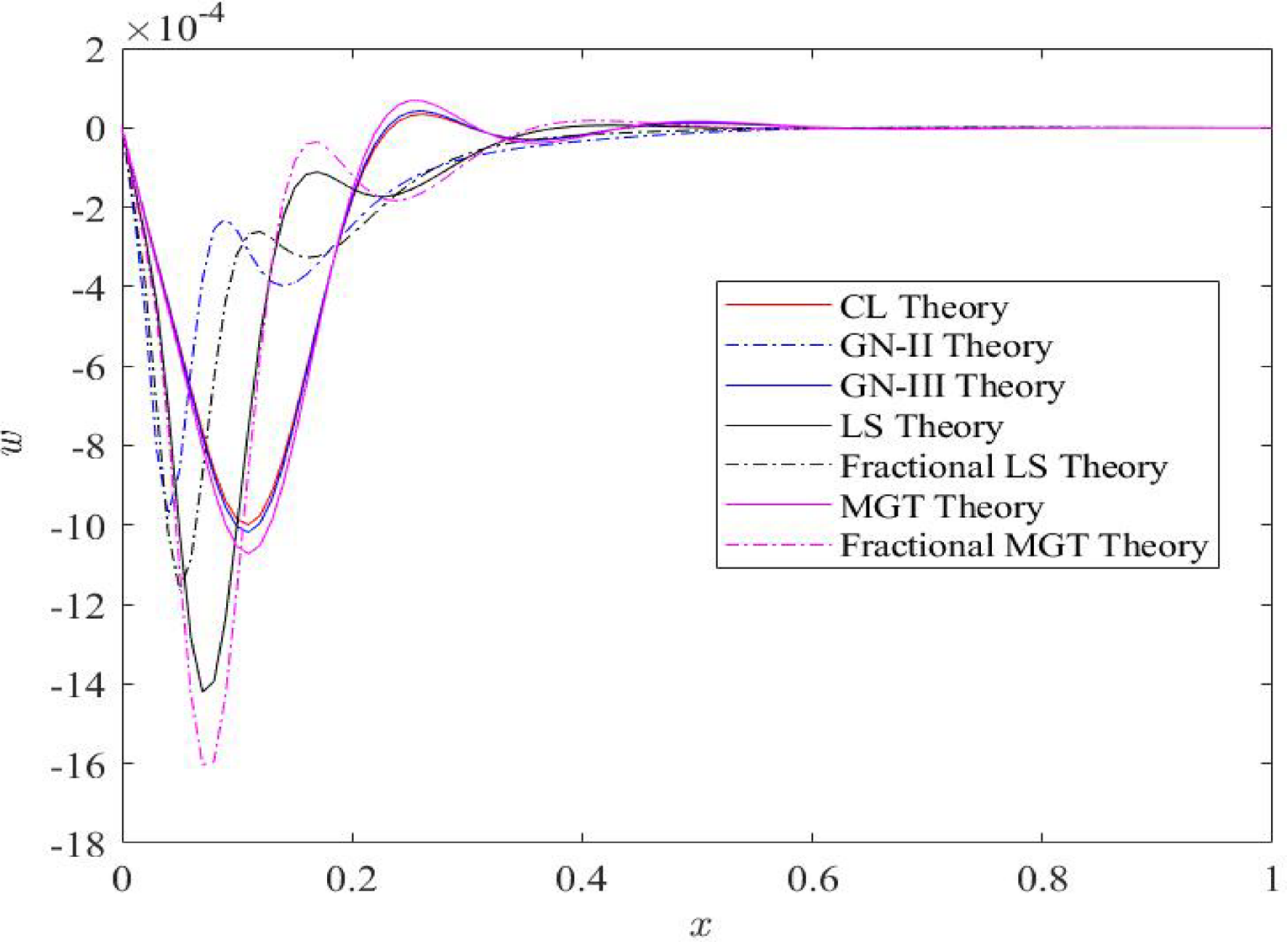
Distributions of lateral deflection $\:w$ field for distinct heat transfer models.

Figures 2, 3, 4 and 5 explore the distributions of dimensionless temperature, displacement, thermal moment and lateral deflection along the beam axis with length 1. Figure 2 demonstrates the nature of temperature distributions along the beam length. This figure exhibits significant features. At the left boundary of beam i.e.𝑥 = 0, the temperature field attains its maximum numerical value that reflects the impact of ramp type heat applied on the left boundary of beam. Behavior of the temperature field does not alter on changing the heat transport model. However the numerical values of the temperature profiles change as the thermal heat conduction model is changed. Further, one can observe that the presence of conventional integer order derivatives, each thermo-elastic theory- classical theory (CL), LS model, GN-II, GN-III models and MGT model speculate the higher numerical values of temperature profiles whereas the profiles of temperature are suppressed and show smaller values in the presence of the fractional order derivatives in the place of integer order derivatives. This is the significance of fractional order thermo-elastic theory of heat transport. Apart from this, CL and LS models present the higher values of temperature whereas MGT and GN-II models depict lower values of temperature field compared to CL and LS, GN-III models. Later on, it is observed that moving towards the right boundary i.e. 𝑥 = 1, profiles of the temperature decay rapidly and become zero near 𝑥 = 0.15. It implies that the curves of the temperature distributions decay very early near the first boundary which indicates that the effects of thermal load are reduced on thermo-elastic waves inside the Nano beam after crossing the distance 𝑥 = 0.15. Higher values of temperature generate the higher stress inside the system that could become a good reason of structural failure of the system. Therefore, it is concluded that MGT model and GN-II model including fractional derivatives are found to be most efficient and accurate models of heat transport to determine the precise and optimum results.

Figure 3 states the nature of displacement field along the axis of beam. Each profile of displacement field starts from a finite value at the left boundary 𝑥 = 0 of Euler beam, then they reach to the peak values rapidly nearer to the left boundary at 𝑥 = 0.1 due to the impact of thermal load on the left boundary. After suffering from many jumps during the journey from the left to the right boundary of beam, displacement plots fade and move to zero nearer to the mid of the axis of Nano beam. Since, displacement field *u* is mathematically described as *u* = − z∂w/*∂x*. This relation conveys that displacement field emerges from the beam’s curvature where the negative numerical values of displacement *u* near the left boundary stipulate compression in that region, as the derivative *dw*/*dx* reflects positive values. On the other hand, the positive values of displacement field indicate tension. In our study, positive values of displacement profiles are obtained closer to the left boundary compared to the negative values of the field. Pattern of plots remain unaffected from the alteration of the heat transfer model while the numerical values of displacement field are changed as the heat conduction model changes. In this case, fractional heat transfer models reveal higher numerical values compared to heat conduction model with traditional integer order derivatives. Like the temperature, displacement graphs convey the lower numerical values for MGT model and GN-II model compared to the CL model, LS model and GN-III model of heat transfer.

Figure 4 communicates the distributions of the bending moment (𝑀) along the length of the beam. The maximum numerical values of bending moment 𝑀 are attained near the left boundary at 𝑥 = 0. Later on bending moment plots are diminished one move towards the right boundary of beam. Transformation from positive to negative numerical values of the bending moment 𝑀 speculates the change in curvature and the bending moment 𝑀 is affected by many factors, including curvature (which is expressed as the second derivative of deflection, 𝜕^2^𝑤/𝜕𝑥^2^), thermal effects ($\:\psi\:$), and non-locality ($\:\xi\:$). Presence of fractional derivatives in thermal conduction model decreases the curvature and consequently reduces the positive numerical values of thermal/bending moment while fractional derivatives enhance the negative numerical values of thermal moment compared to the integer order derivatives. MGT model and GN-II modes predict the smallest positive numerical values of thermal moment. The positive thermal moment 𝑀 near the left boundary of beam reveals sagging, whereas the negative thermal moment near the right boundary of beam 𝑥 = 1 is known as hogging. This figure presents the presence of the maximum positive moment close to the left boundary of the beam.

Figure 5 depicts the deflection field (𝑤) along the beam-axis 𝑥 ranging from 0 to 1. Deflection field exhibits zero numerical value at both the left and right boundary of the beam. This behavior satisfies the boundary conditions of our study. Plots of deflection field move towards the negative values as one go from the left boundary to the right boundary of beam. After facing several jumps during their journey, deflection profiles disappear ultimately neat the right boundary. Inclusion of the fractional derivatives in heat conduction model suppresses the domain of influence of the field and results the lowering of the numerical values. Moore Gibson Thompson model (MGT model) depicts the smallest numerical values of deflection compared to the CL, LS, GN-II, and GN-III models of heat transfer. From the figure, it is noted that deflection curve achieves 5% less values of deflection quantity compared to the LS theory of heat transfer and around 8% less values from the CL theory of heat transport. However this margin becomes higher for the case of displacement distributions. Similar type results were reported by Tiwari et al.^16^. Maximum values of deflection profiles are achieved near the left boundary of beam which clearly shows the impact of ramp type static heat at the left boundary.

Research findings indicated above coincide with the prior investigations such as Hosseini et al.^42^ where the results were compared LS and CL thermoelastic models for beam at Nano scale where he proved that quantitative values of the physical fields are reduced for LS model compared to the CL model of heat transfer. Alrubea and Abouelregal^38^ reported in their research article of Nano scale beam that the field quantities like temperature, displacement, bending moment and thermal stress obtain highest values under the purview of CL model of heat transfer. This result evidently presents similarity with the results of our study.

### Influences of fractional quantity $\:\boldsymbol{\alpha\:}$ on the behaviour of physical fields

In this subsection, all the physical fields- temperature, displacement, bending moment and lateral deflection are depicted for distinct values of the fractional parameter $\:\alpha\:$ = 0, 0.05, 0.5, 1. Graphical results are derived for Moore Gibson Thompson heat transfer theory (MGT). For the purpose of plotting the graphs, following numerical values of parameters are taken into account-$\:{\tau\:}_{q}=0.02$, $\:t=0.25$, $\:{t}_{0}=0.2$, $\:v=0.3$, $\:\xi\:=0.003$.

**Fig. 6:**
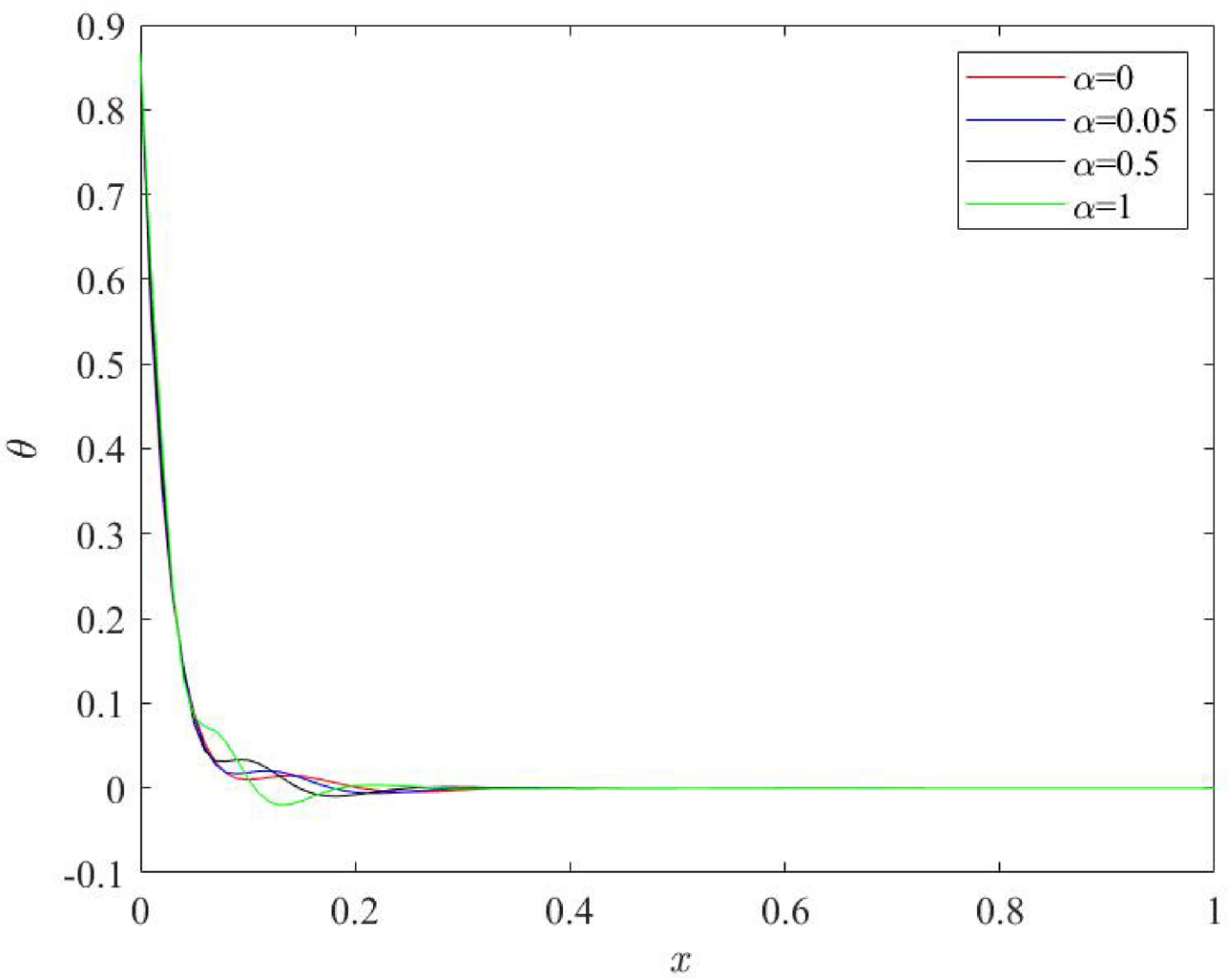
Behaviour of temperature profile $\:\theta\:$ for various numerical values of fractional quantity $\:\alpha\:$.

**Fig. 7:**
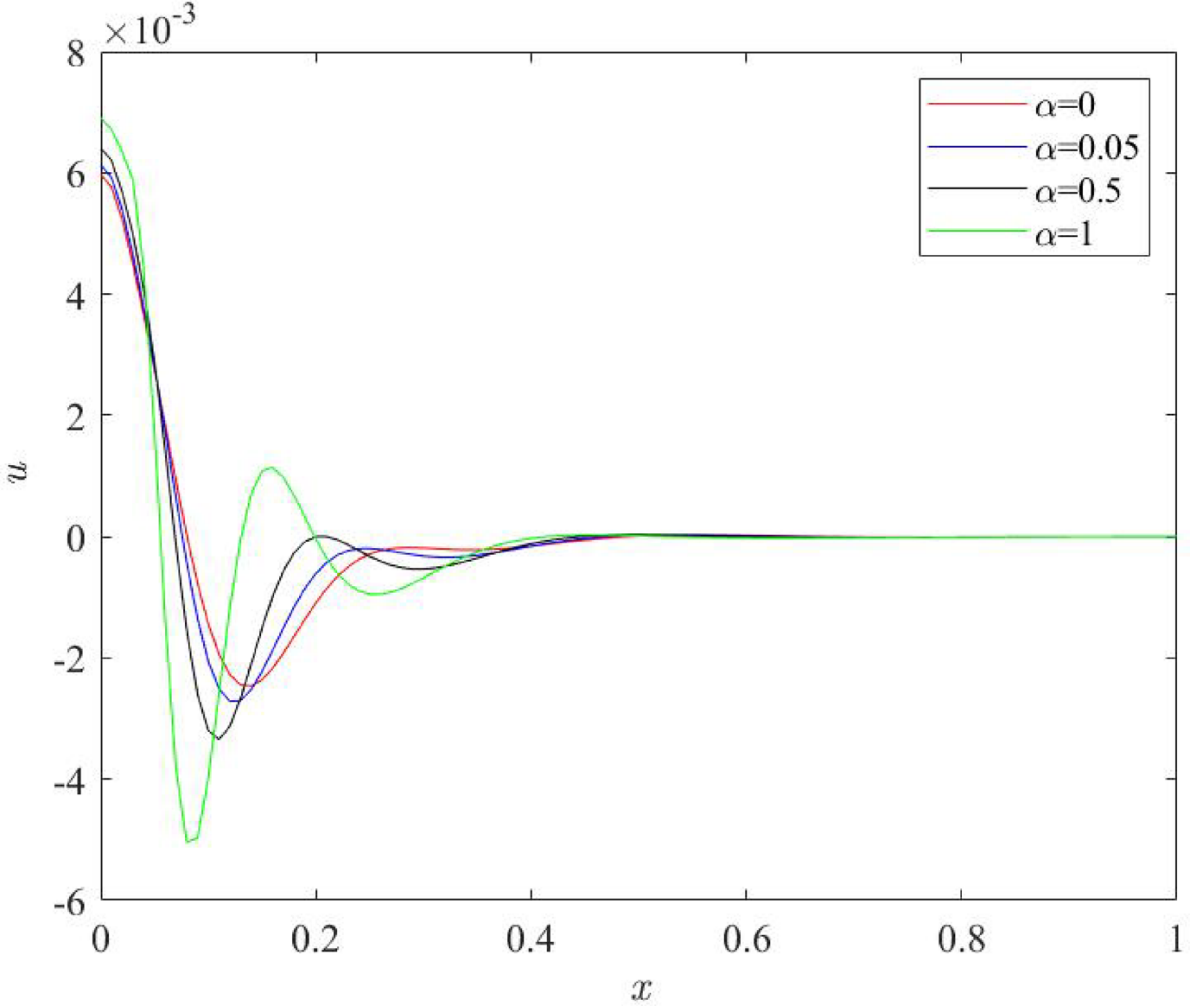
Behaviour of displacement profile $\:u\:$for various numerical values of fractional quantity $\:\alpha\:$.

**Fig. 8:**
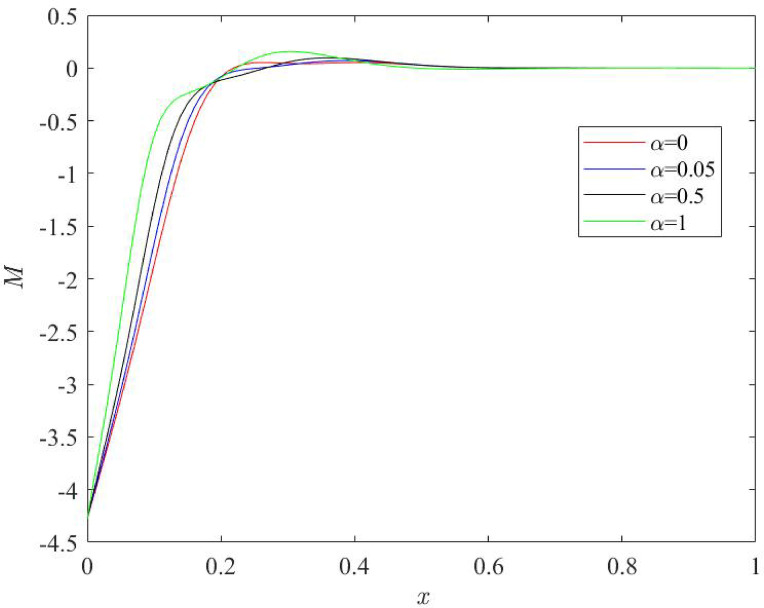
Behaviour of thermal moment profile $\:M$ for various numerical values of fractional quantity $\:\alpha\:$.

**Fig. 9:**
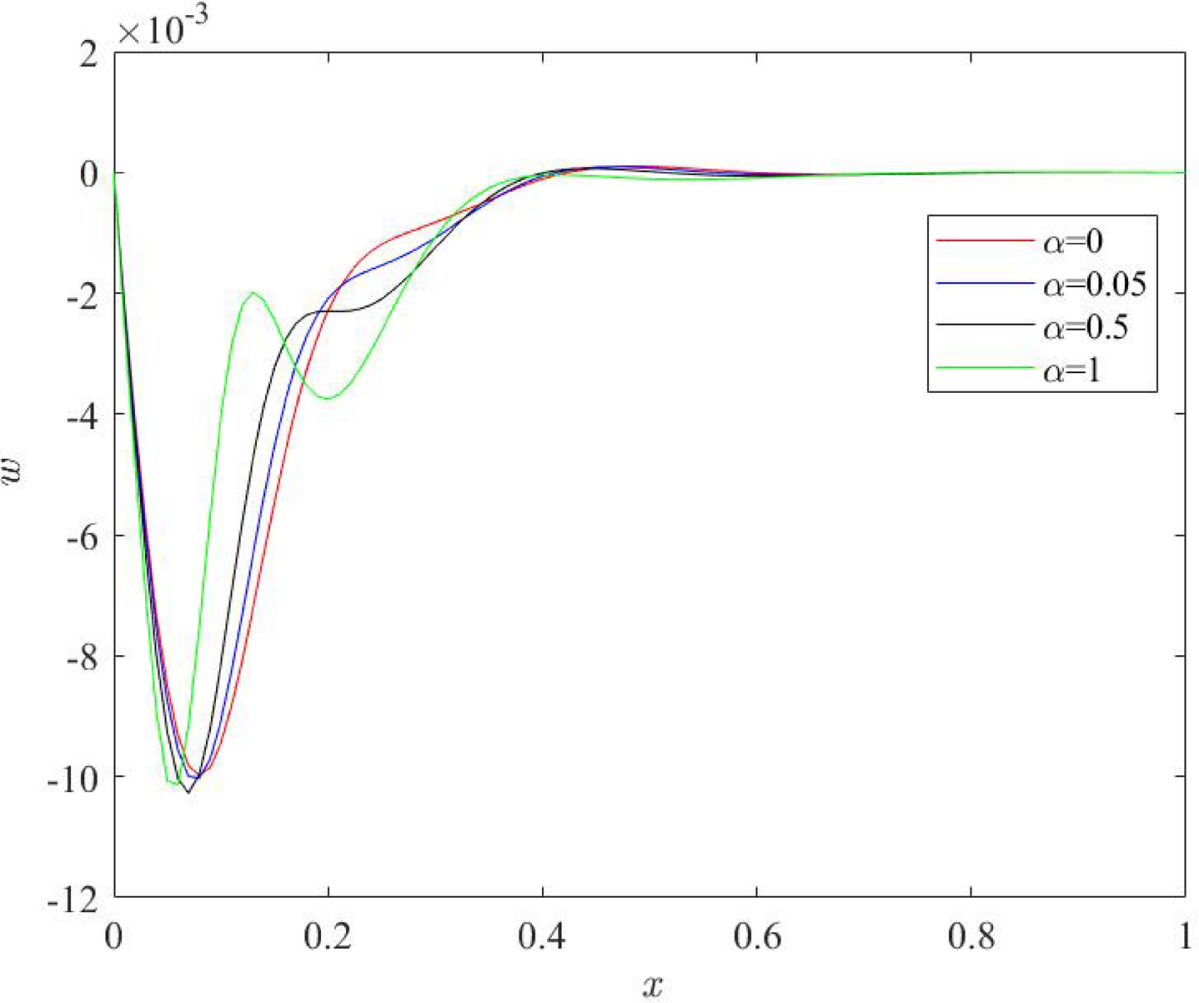
Behaviour of lateral deflection profile $\:w$ for various numerical values of fractional quantity $\:\alpha\:$.

Figures 6, 7, 8 and 9 stipulate a thorough analysis of the Nano beam, focusing on the impact of fractional parameter $\:\alpha\:$ on dimensionless field quantities - temperature (*θ*), displacement (*u*), bending moment (*M*) and lateral deflection (*w*). Numerical values of fractional parameter $\:\alpha\:$ are considered as: 0, 0.05, 0.5, 1. The case when $\:\alpha\:$ = 0 predicts the traditional Moore Gibson heat transfer model (MGT) where fractional derivatives are replaced by integer order derivatives. Further, $\:\alpha\:$ = 0.05 indicates the case closer to traditional MGT heat transfer model.

As speculated in Fig. 6, impacts of fractional quantity $\:\alpha\:$ are displayed on the profiles of temperature field. Highest values of temperature plots are obtained at the left boundary of beam $\:x=0$ which exhibits the direct impact of ramp type heat applied on the left boundary. Then, plots start to decrease as one move from the left to the right boundary $\:x=0.$ This nature of temperature field exhibits the stable characteristics of heat transmission inside the beam which is due to the suitable heat transfer theory i.e. MGT model of heat transfer. Variation pattern of temperature does not alter for different numerical values of fractional parameter $\:\alpha\:$ whereas numerical values are clearly affected as fractional parameter $\:\alpha\:$ changes. It is noticed that when $\:\alpha\:$ increase, temperature plots decay earlier toward zero value.

Figure 7 conveys the nature of displacement distributions for various numerical values of fractional quantity $\:\alpha\:$. Profiles of the displacement field begin from a finite positive value at the left boundary of the Nano beam $\:x=0$; then they rapidly decrease and predict negative numerical values. Later on, after facing jumps, they disappear towards the right boundary of beam $\:x=1.$ Magnitude of displacement field prominently enhances towards the negative direction while slightly increases towards the positive direction. For the greatest numerical value of fractional quantity i.e. $\:\alpha\:=1$, this behaviour of displacement is most prominent. In this way, for higher $\:\alpha\:$, displacement profile indicates lower values as plots move towards negative direction prominently compared to the positive direction. This nature of plots reveals the decrease frequency shift noise in the nano beam and consequently enhances the sensitivity of the beam.

Figure 8 characterizes the variations of thermal moment for different fractional quantity $\:\alpha\:.$ Plots of bending moment start from negative value at the left boundary $\:x=0$. Afterwards, they move towards the positive direction as one go towards the right boundary and finally, they disappear at the mid-point $\:x=0.5$ of nano beam. Trend of variation is observed to be unaffected for different numerical value of fractional quantity $\:\alpha\:\:$but numerical values of the thermal moment are altered when the numerical value of fractional quantity $\:\alpha\:$ is changed. Magnitude of numerical values of thermal moment becomes smaller for higher values of fractional parameter. Smaller thermal moment indicate the reduced stress and enhances the quality of the system. Zenkur and Abouelregal^39^ indicated in their study that higher values of fractional quantity reduce the quantitative values of thermal moment. Therefore, it is noted that our investigation exhibits similarity with the previous studies.

As stipulated in Fig. 9, mode of variation of lateral deflection plots remains unaffected from the changes in fractional quantity $\:\alpha\:$ while numerical values of deflection $\:w\:$ are noticed to be significantly affected as $\:\alpha\:$ changes. Deflection profiles indicate zero value at left and right boundary of beam which satisfies the boundary conditions opted in the current study. Highest values of deflection occur near the first boundary of the system for $\:0<x<0.1$. This is due to the presence of ramp heat at the first boundary of nano beam. Apart from this, magnitude of lateral deflection reveals lower numerical values for greater value of fractional parameter $\:\alpha\:.$ Lateral deflection is directly proportional do the bending stress. Lower deflection originates the lower values of bending stress and increases the sensitivity, functioning and life of the system. Moreover, it is noted that curves of each physical field – temperature, stress, bending moment and deflection diverge before the min point of Nano scale beam which exhibits that the impact of thermal load applied on the initial boundary of beam does not remain prompt after crossing the midpoint of the beam.

### Influences of non-local quantity $\:\xi\:$ on the behaviour of physical fields

**Fig. 10:**
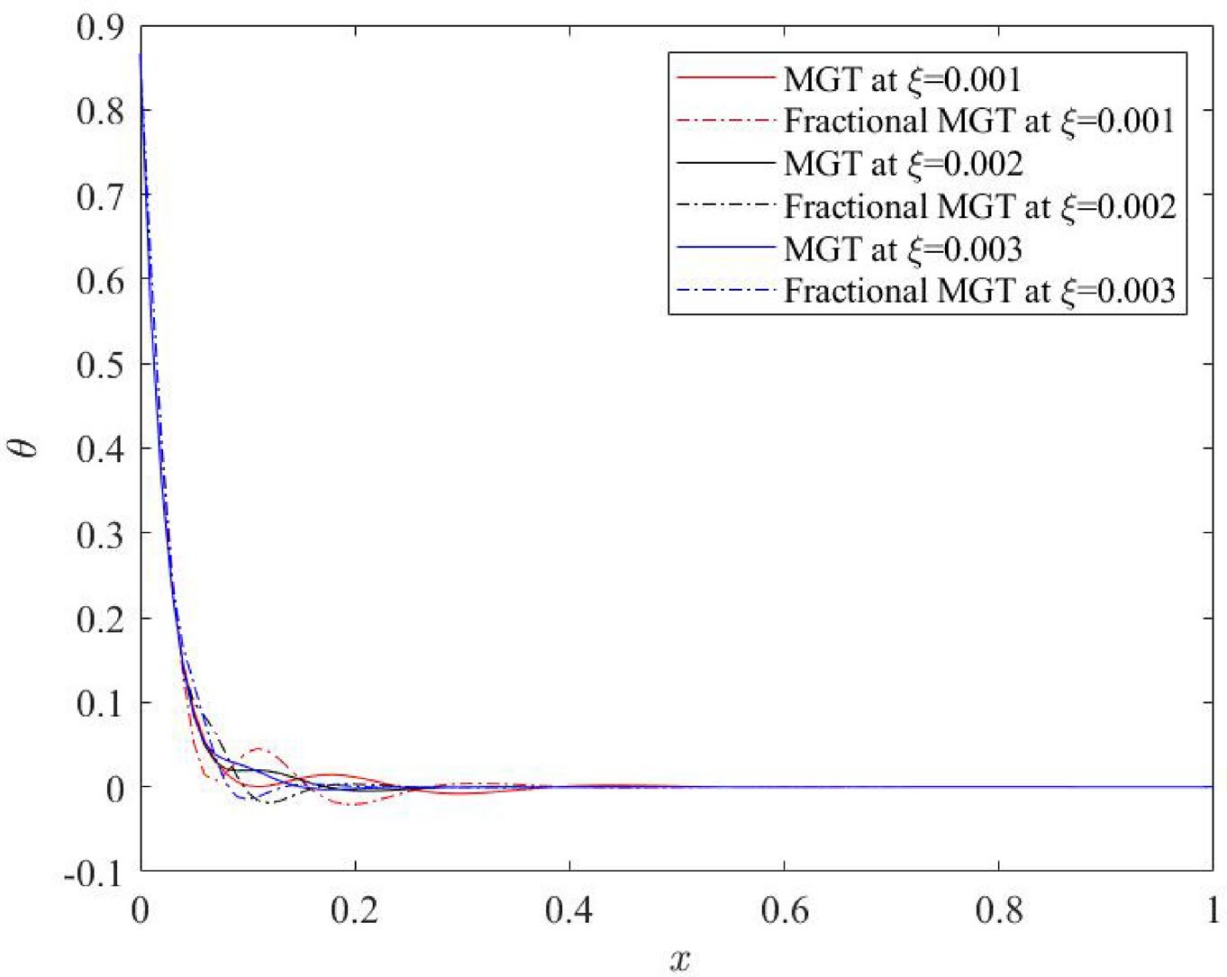
Temperature profile $\:\theta\:$ for various numerical values of non-local quantity $\:\xi\:$.

**Fig. 11:**
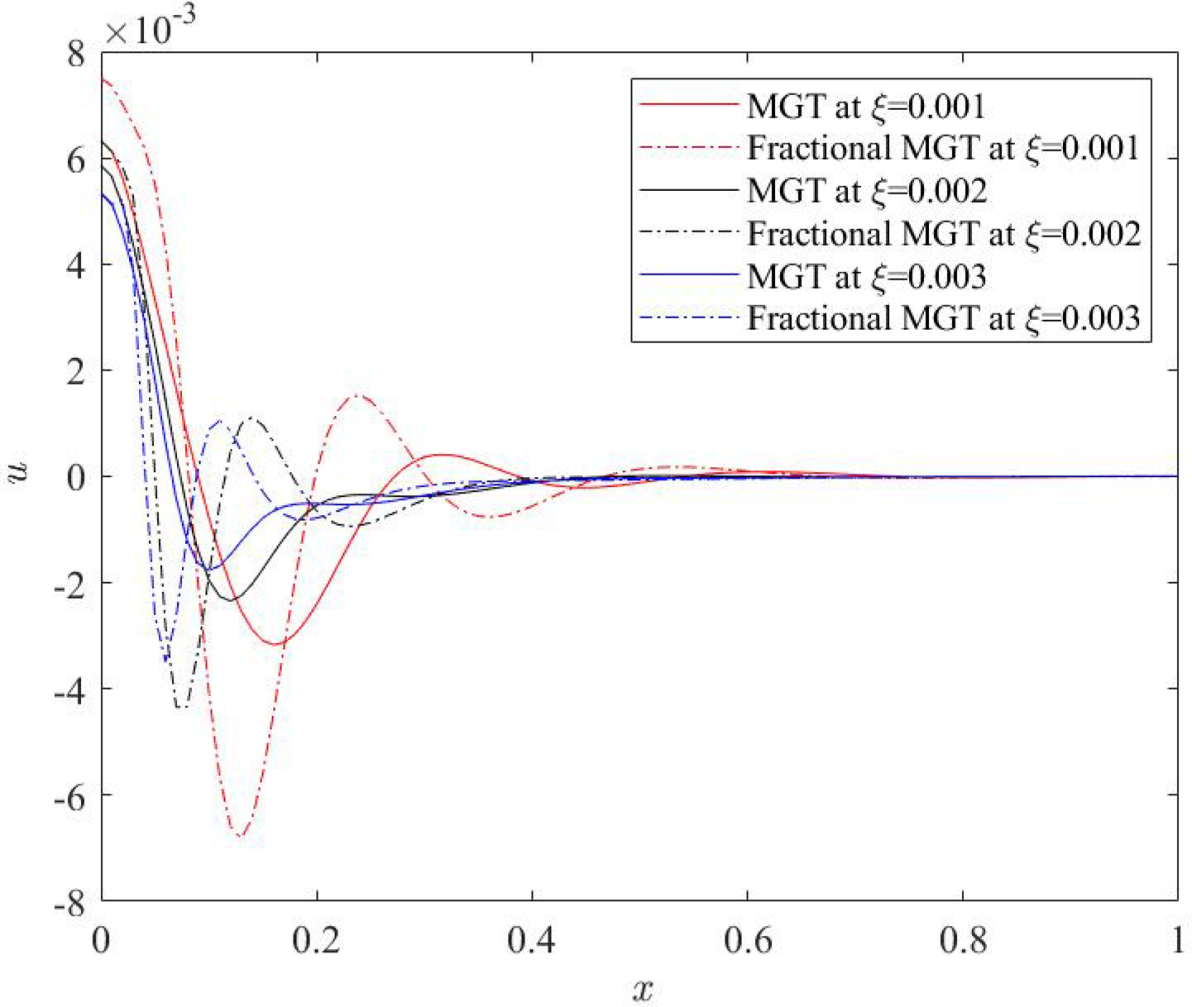
Displacement profile $\:u$ for various numerical values of non-local quantity $\:\xi\:$.

**Fig. 12:**
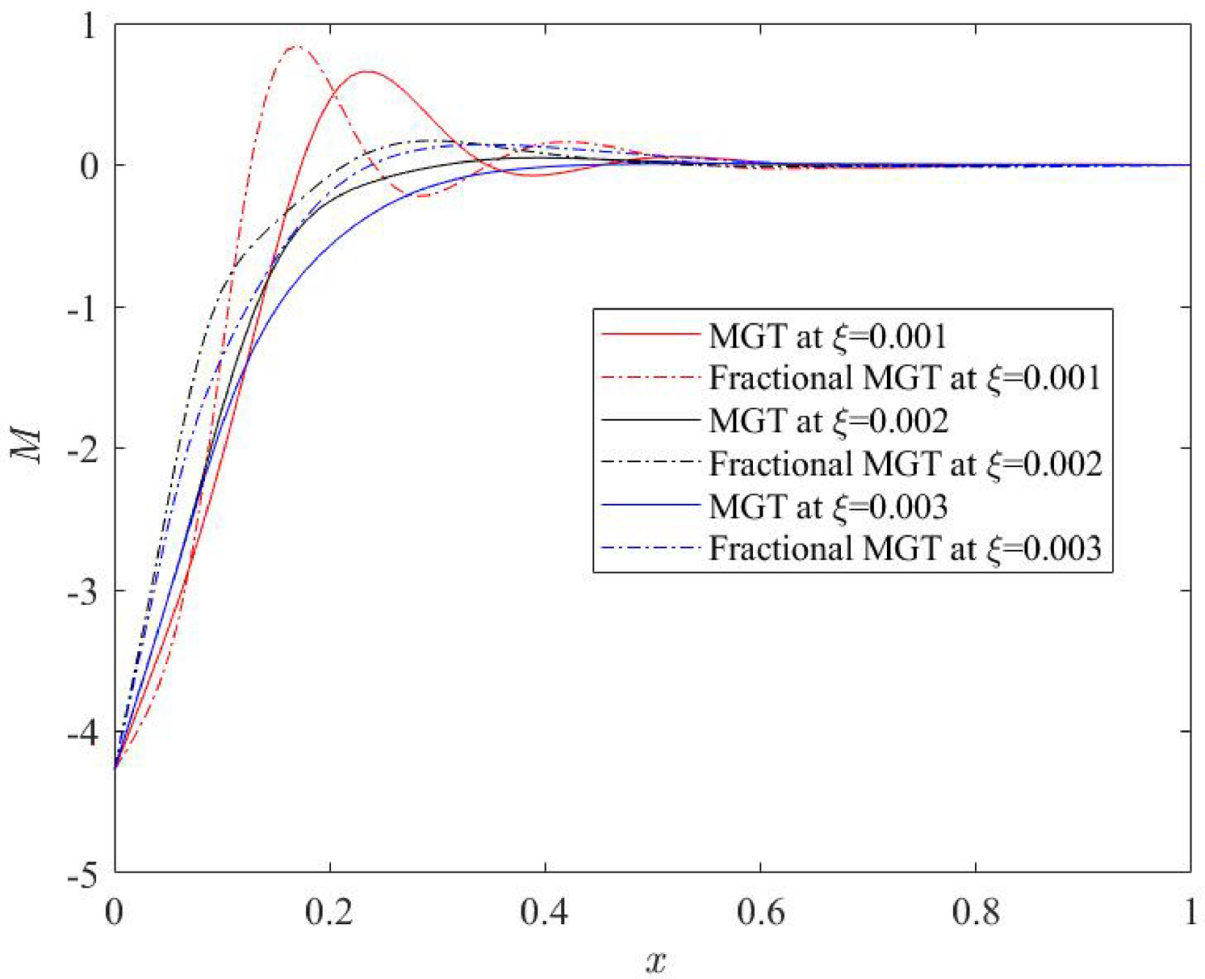
Thermal moment profile $\:M\:$for various numerical values of non-local quantity $\:\xi\:$.

**Fig. 13:**
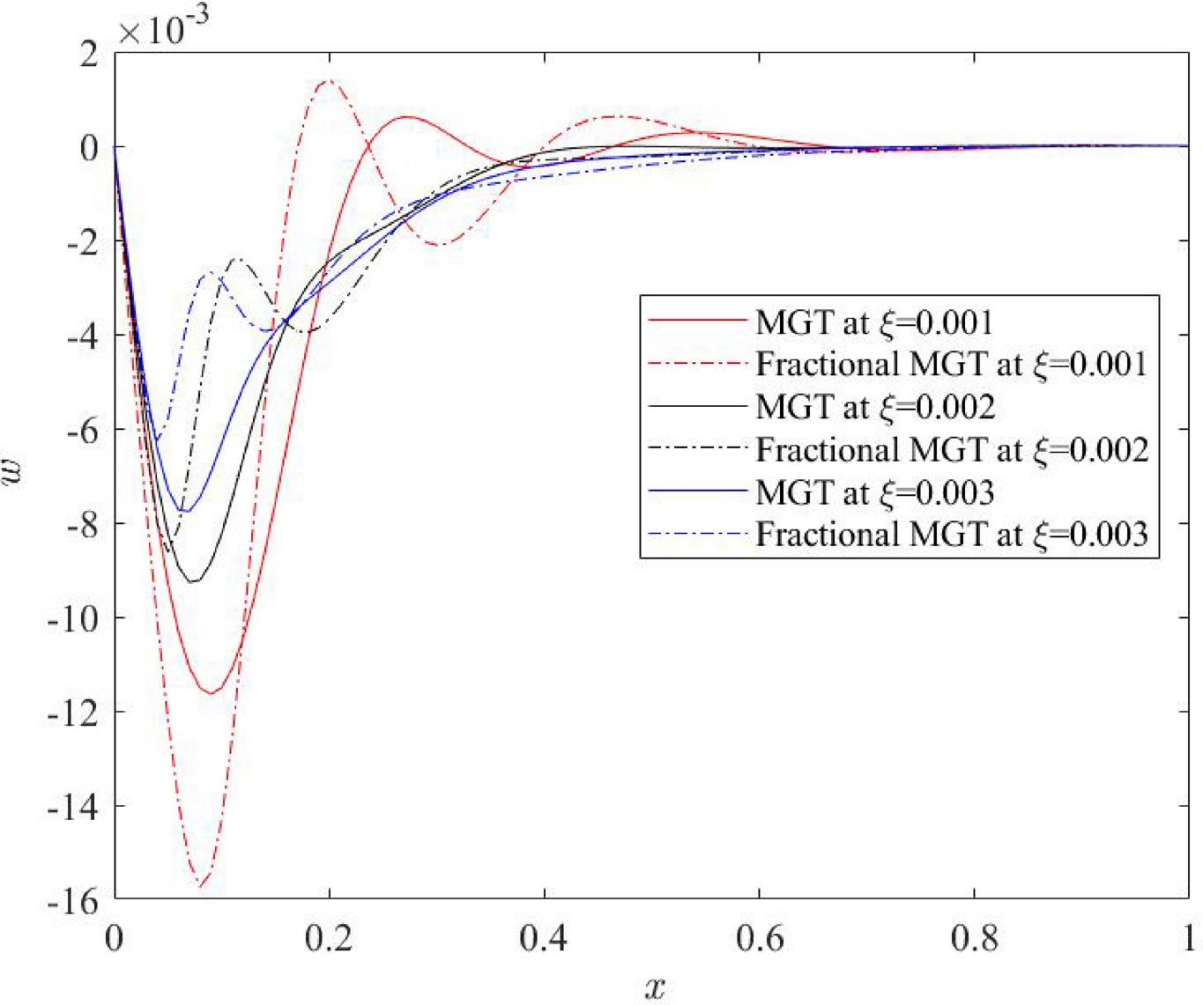
Lateral deflection $\:w\:$profile for various numerical values of non-local quantity $\:\xi\:$.

Current subsection investigates the effects of the nonlocal parameter 𝜉 on the dimensionless physical fields—temperature, displacement, moment and lateral deflection for fractional MGT model and conventional MGT model as represented in Figs. 10, 11, 12 and 13 respectively. Numerical values of 𝜉 are considered as: 𝜉 = 0.001, 𝜉 = 0.002, 𝜉 = 0.003. Other parameters are taken as: $\:v=0.3$, 𝑡 = 0.25, $\:{t}_{0}$ = 0.2, 𝜏_𝑞_ = 0.02.

Effects of nonlocal quantity $\:\xi\:$ are depicted on the profiles of temperature distributions under the purview of MGT heat transfer model in the presence and absence of fractional derivatives in Fig. 10. Trend of variation of plots are noticed to be unaffected from the distinct values of nonlocal parameter $\:\xi\:$. But the numerical values of temperature profiles are observed to be prominently affected as the numerical value of nonlocal quantity changes. Maximum temperature is attained at the left boundary $\:\left(x=0\right)\:$of beam which explicitly shows the influence of ramp type heat source applied on the left boundary. Later, profiles of temperature start to decrease and vanish finally. It is noted that temperature plots decay earlier for smaller values of nonlocal quantity for both fractional MGT model and traditional MGT model. Further, plots face more jumps under the influence of fractional derivatives compared to the MGT model without fractional derivative.

Figure 11 represents the behaviour of displacement field for distinct numerical values of nonlocal quantity $\:\xi\:$. Displacement plots are found to be significantly affected as the numerical value of nonlocal quantity is changed. One can notice that greater numerical value of nonlocal quantity suppress the domain of the displacement field and consequently smaller numerical values of the displacement field are obtained. This result is highly useful in physical world. Smaller values of the displacement profiles indicate high linearity, low energy dissipation, lower stress and higher sensitivity of the sensors.

Figures 12 and 13 indicate the nature of thermal moment and lateral deflection, respectively for different nonlocal parameter $\:\xi\:.$ Similar to the displacement field, thermal moment and deflection are significantly influenced from nonlocal quantity. One can notice that for small values of nonlocal parameter, curves of thermal moment and deflection exhibit very higher values on jumps near the left boundary. This is also due to the presence of heat input on the left boundary. Whereas the curves of both field quantities show finite and stable characteristics for higher numerical values of nonlocal parameter.

Therefore, it is concluded from the above plots that the nonlocal parameter affects the thermo-mechanical waves very prominently however after crossing the mid distance, all curves fade and tend to zero value. Greater value of nonlocal quantity predicts the stable and smooth behaviour of the field quantity as increasing the numerical values of nonlocal quantity suppresses the domain of influence of physical fields and reduce the numerical values. As a result, low energy dissipation occurs inside the system that indicates the high sensitivity and better outcomes of the system.

### Influences of ramp type parameter $\:{\boldsymbol{t}}_{0}$ on field quantities under fractional MGT model and conventional MGT model without fractional derivatives

This section is aimed to establish the impact of ramp type parameter $\:{t}_{0}\:$on distributions of temperature, displacement, thermal moment and lateral deflection. Curves are plotted for MGT model with and without fractional derivatives. Quantitative values of $\:{t}_{0}$ are taken as: $\:{t}_{0}$ = 0.01, $\:{t}_{0}$ = 0.1, $\:{t}_{0}$ = 0.5.

**Fig. 14:**
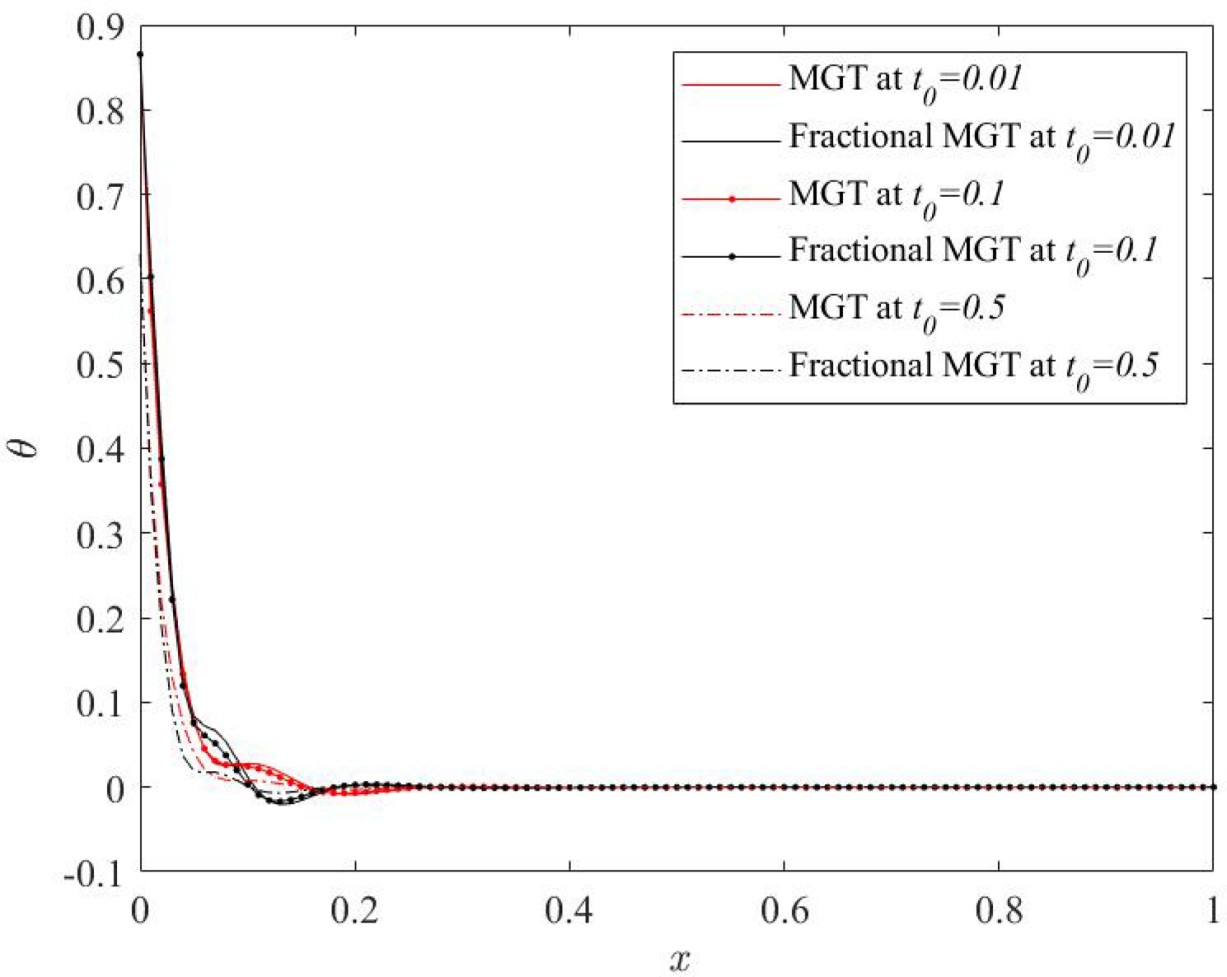
Temperature profile $\:\theta\:\:$for various numerical values of ramp type parameter $\:{t}_{0}$.

**Fig. 15:**
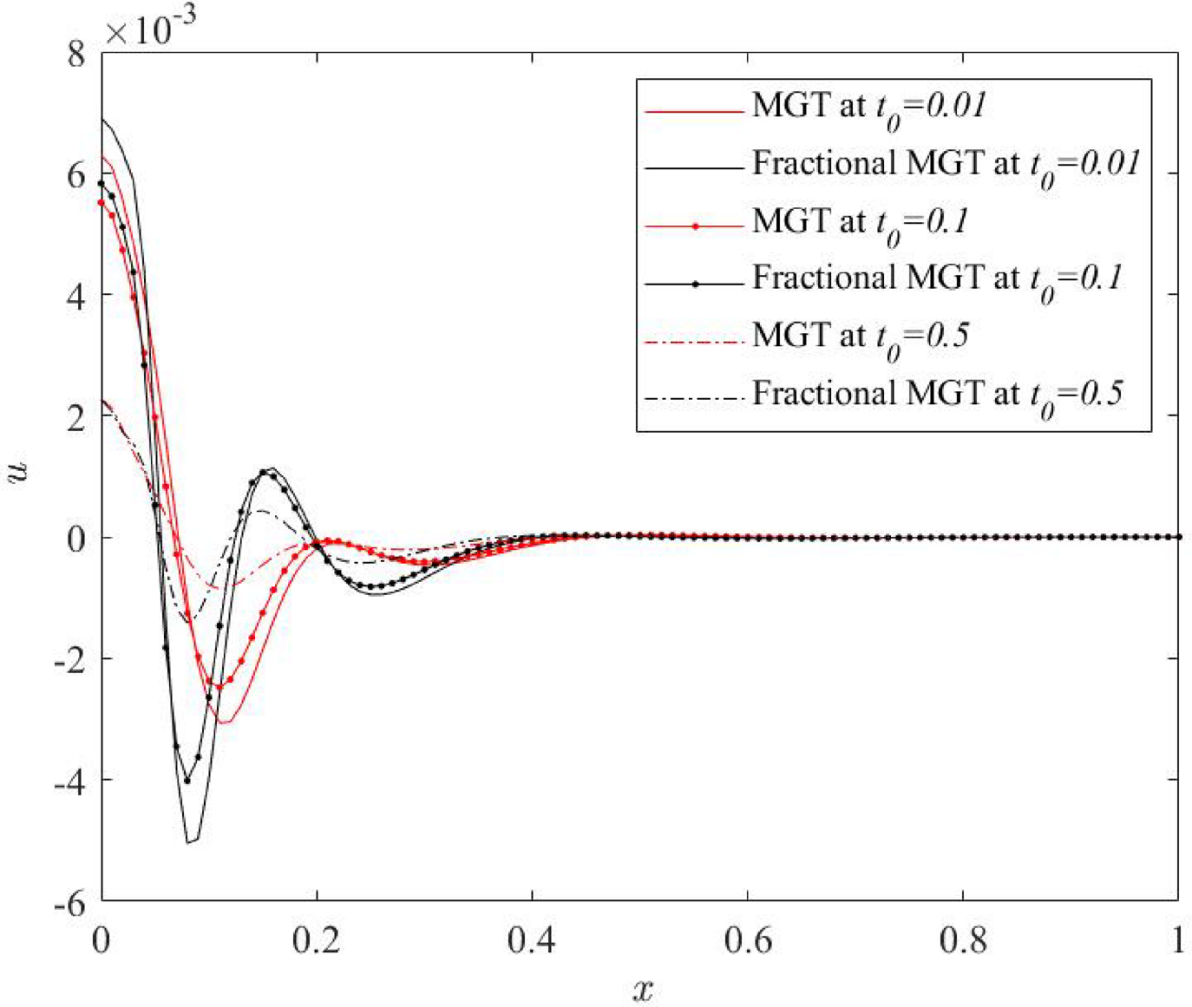
Displacement profile $\:u\:$for various numerical values of ramp type parameter $\:{t}_{0}$.

**Fig. 16:**
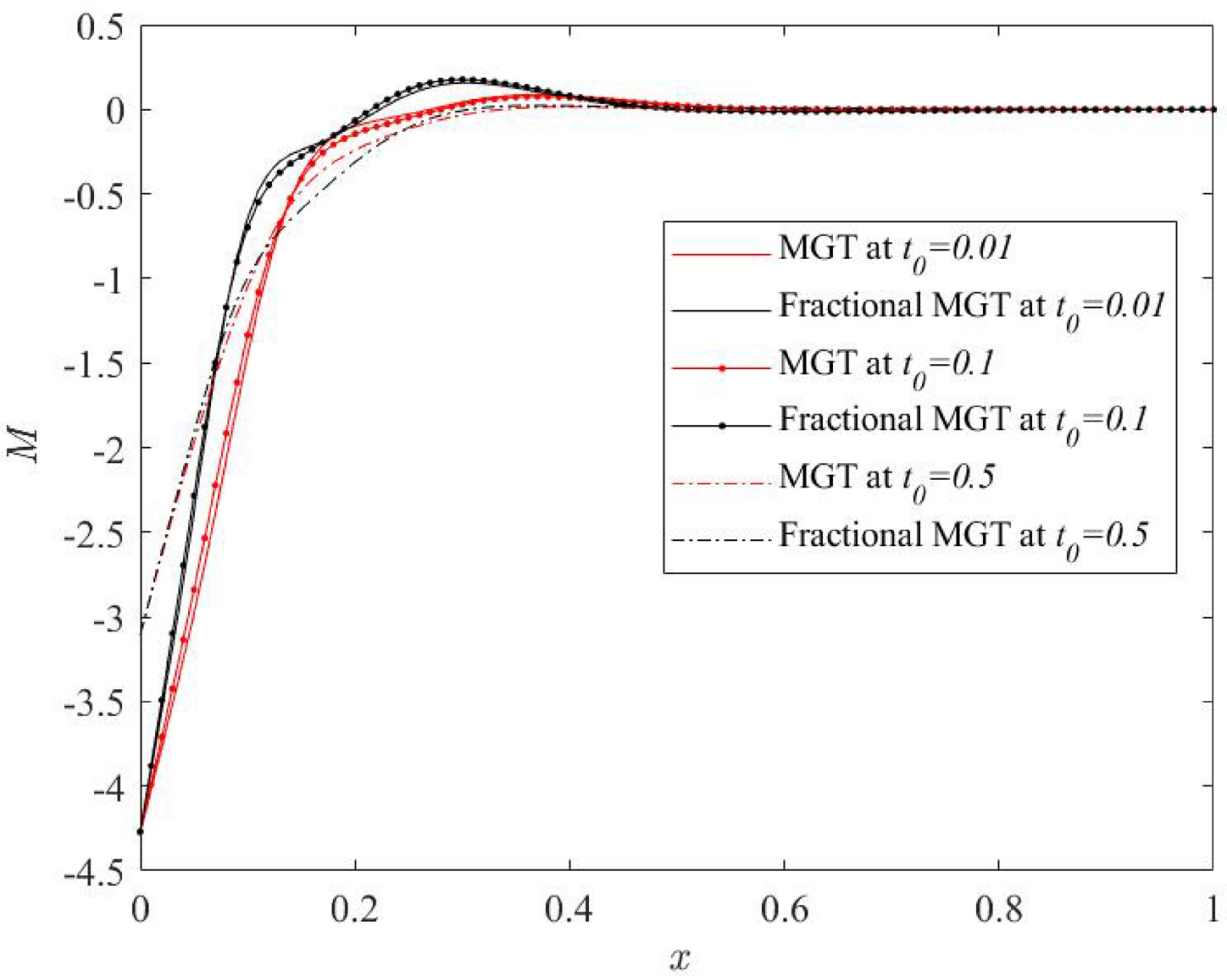
Thermal moment profile $\:M$ for various numerical values of ramp type parameter $\:{t}_{0}$.

**Fig. 17:**
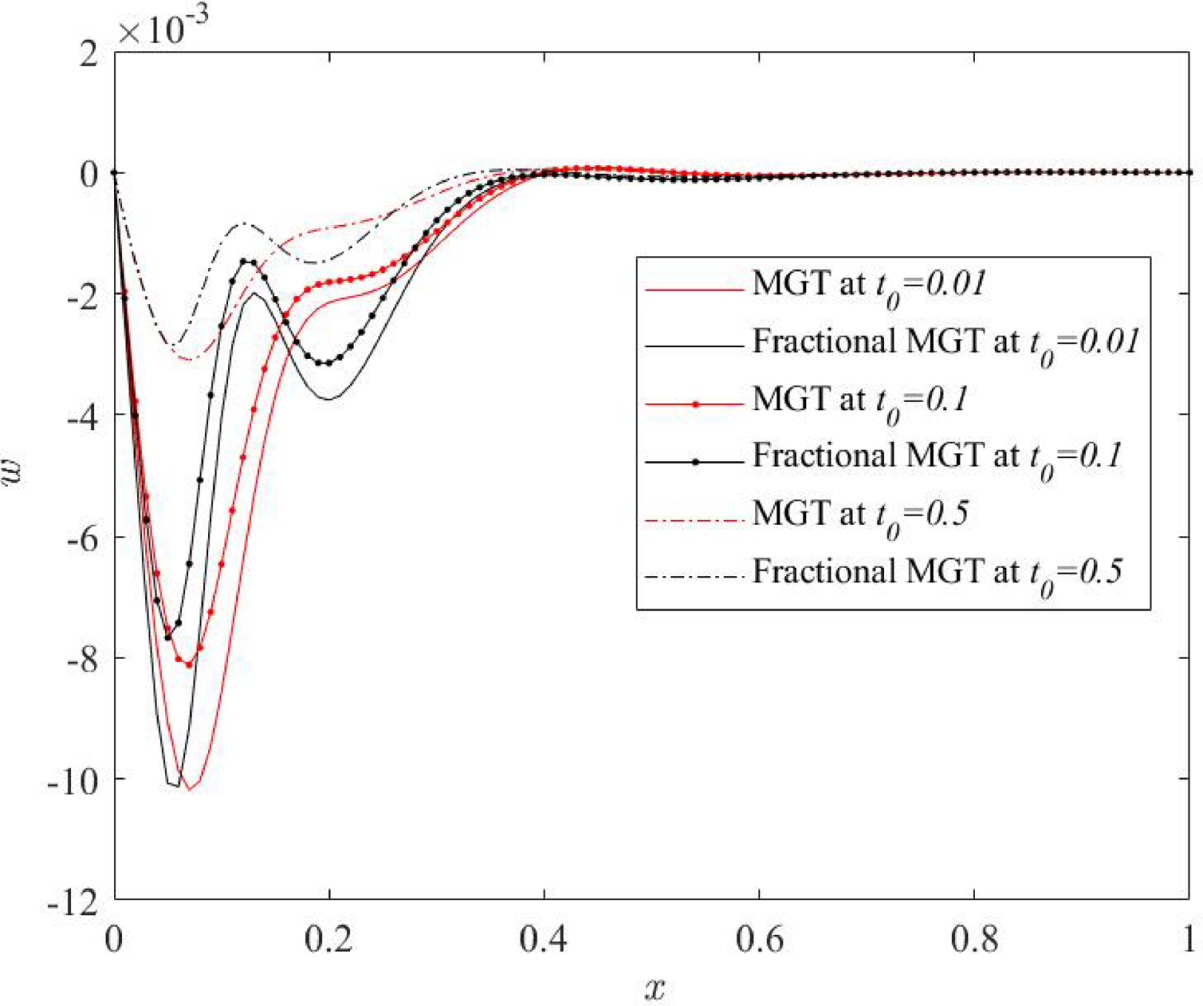
Lateral deflection profile $\:w\:$for various numerical values of ramp type parameter $\:{t}_{0}$.

From Figs. 14, 15, 16 and 17, significant effects of ramp type quantity $\:{t}_{0}$ are perceived on the numerical values of the curves of all physical fields, especially displacement, thermal moment and deflection. Higher value of ramp type quantity reduces the domain of physical fields and reveals smaller values of the field quantities. Apart from this result, it is noticed that the presence of the fractional derivatives suppress the domain of influences of the field distributions.

Hence, it is summarized that greater numerical value of ramp type parameter generates lower dissipation of energy and enhances the sensitivity of the system. Further, it is observed that the presence of fractional derivatives improves the quality of the system as it also helps to lower the thermal stress and enhances the life of the system that can predict the precise and optimum results.

### Influences of velocity of moving heat source on physical fields under the effect of fractional MGT model and traditional MGT model including integer order derivatives

In physical world, several situations are not defined by stationary thermal load. Such situations are accomplished by moving heat source for example scanning laser heating, moving electric current hotspots in Nano electromechanical resonators, Nano-manufacturing and thermal writing etc. Therefore, current investigation attempts to examine the impact of moving thermal load inside the Nano beam system. Motive of this subsection is to reveal a comprehensive analysis of the computational outcomes for the Nano beam made from silicon material, for various numerical values of non-dimensional dynamic load velocity (𝜐) on the dimensionless distributions of temperature (𝜃), displacement (𝑢), thermal moment (𝑀) and lateral deflection (𝑤). Detailed analysis focusing the impact of the velocity of dynamic load is presented for three different values of the velocity 𝜐: 𝜐 = 0.01, 𝜐 = 0.03 and 𝜐 = 0.05.

**Fig. 18:**
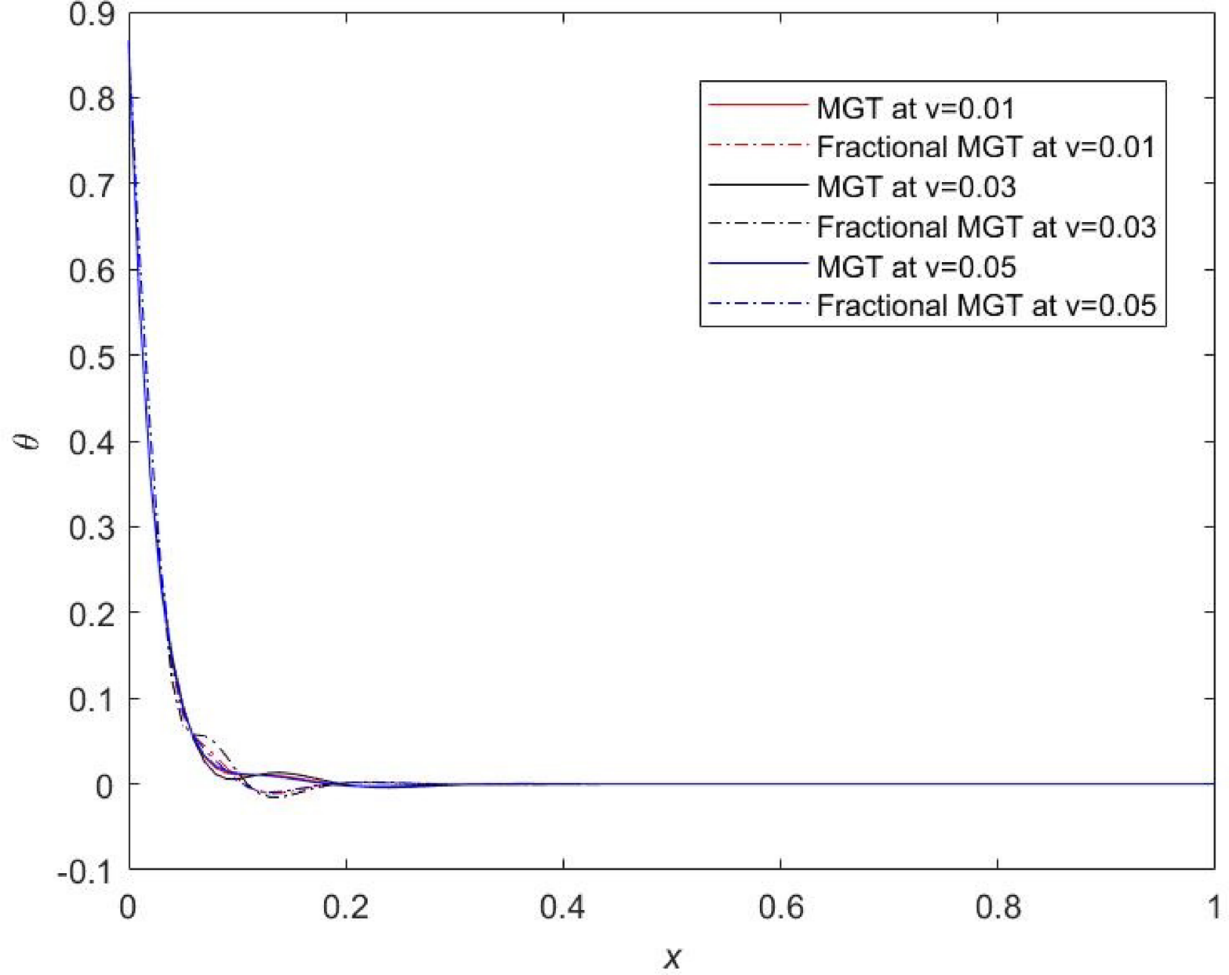
Temperature profile $\:\theta\:$ for distinct numerical values of velocity of dynamic load.

**Fig. 19:**
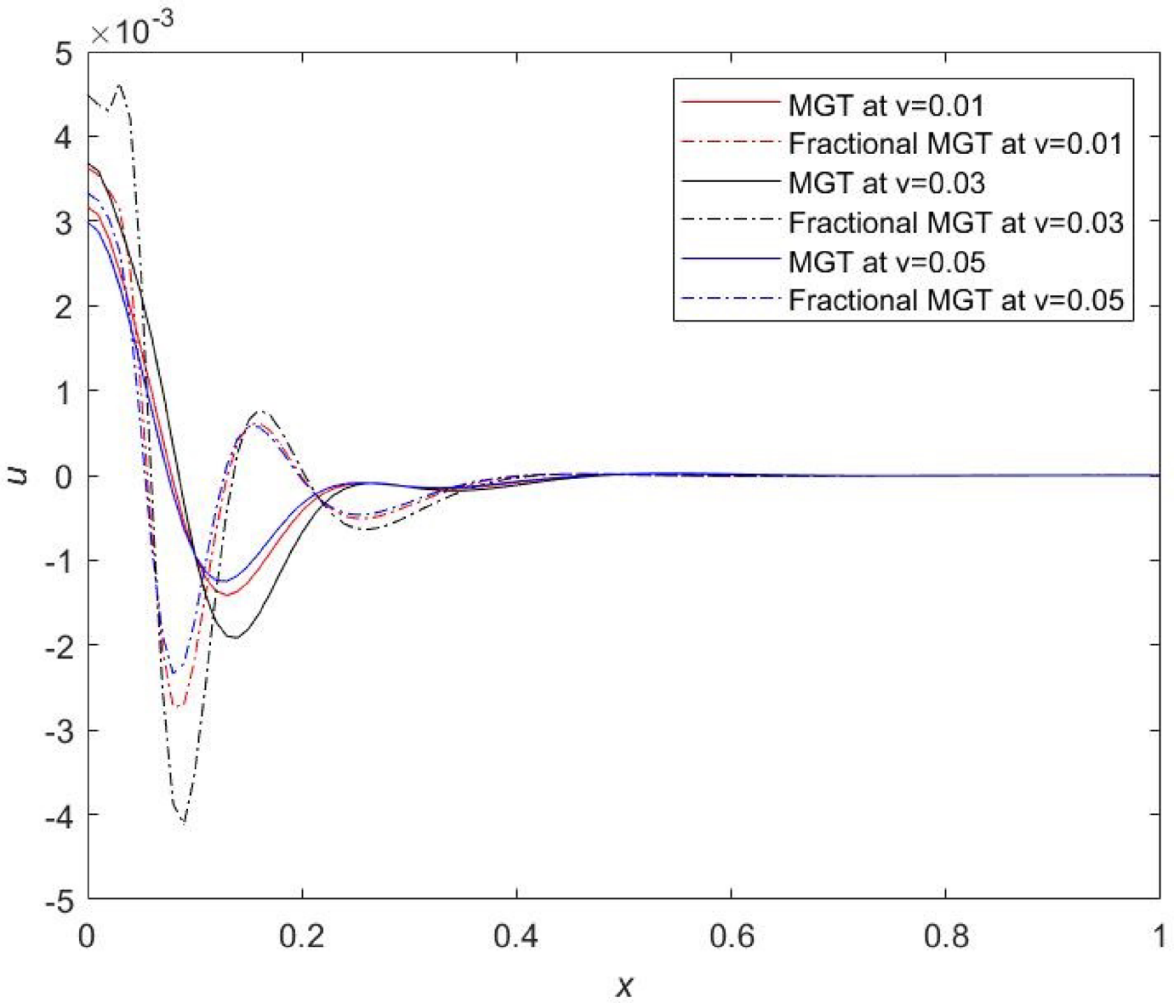
Displacement profile $\:u$ for distinct numerical values of velocity of dynamic load.

**Fig. 20:**
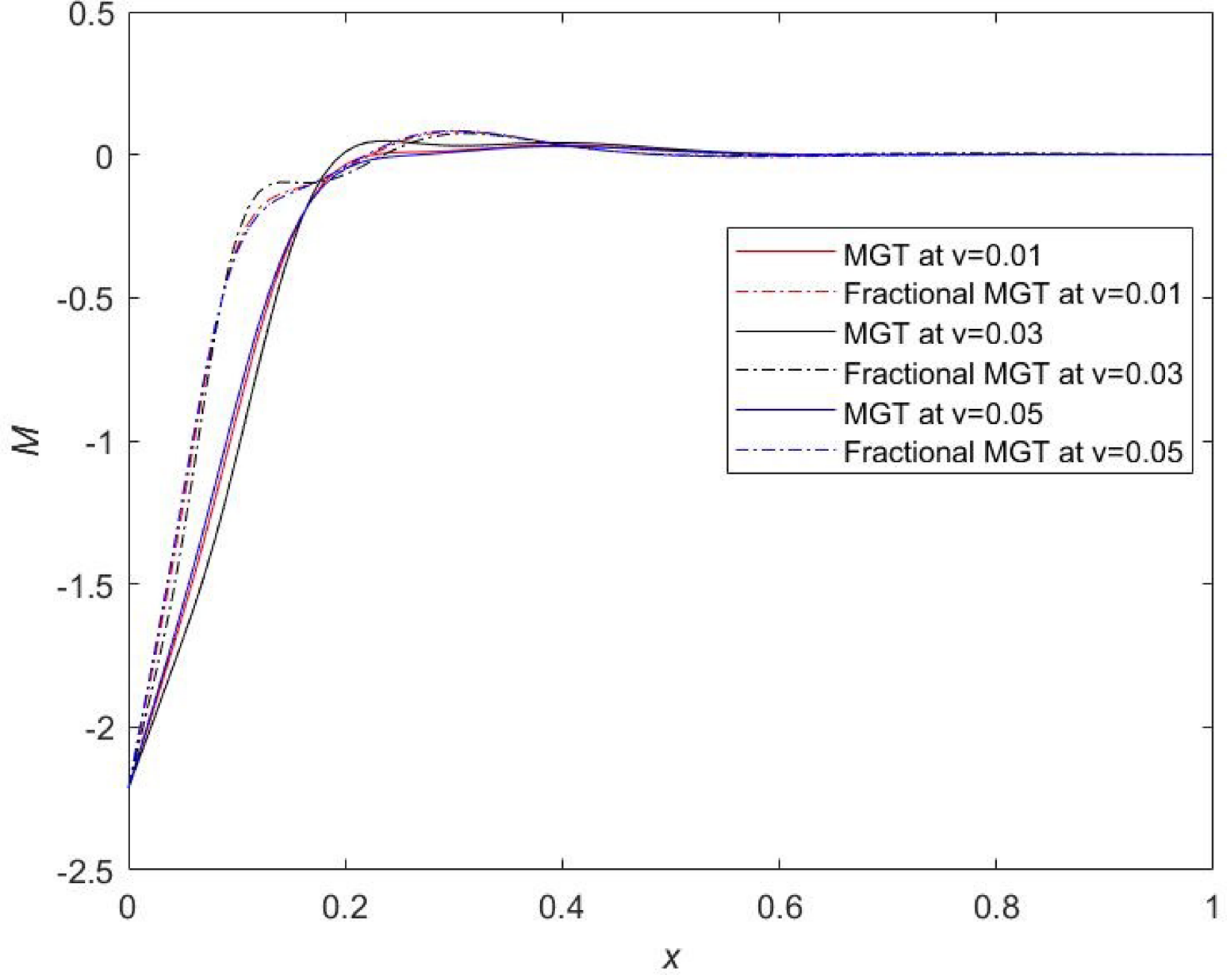
Thermal moment profile $\:M\:$for distinct numerical values of velocity of dynamic load.

**Fig. 21:**
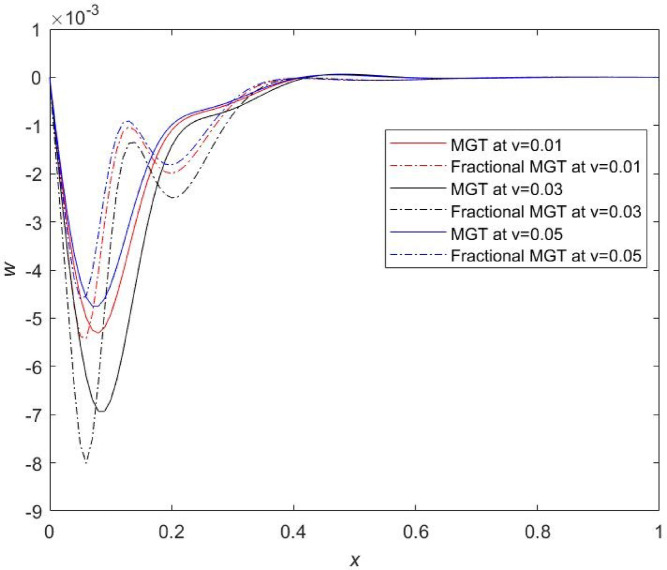
Lateral deflection $\:w\:$ profile for distinct numerical values of velocity of dynamic load.

Figures 18, 19, 20 and 21 examine the impact of the heat source velocity on the variations of curves of temperature, displacement, thermal moment and lateral deflection, respectively. From Fig. 18, it is noted that mode of variation of plots does not alter from the changes in the numerical values of the velocity of moving load while their numerical values are significantly affected from the changes in the velocity. One can observe that for small value of the velocity of moving load, temperature curves indicate smaller numerical values in both fractional MGT model and conventional MGT model of heat conduction. Similar results were reported by Tiwari et al.^16^. This result indicates that smaller values of velocity lowers the dissipation of energy inside the Nano beam system and enhance the life of the system. Aldandani and Abouelregal^43^ and Alrubia and Abouelregal^38^ investigated that the temperature field achieves higher numerical values for the higher velocities of the dynamic load in the framework of generalized heat transport model including phase lags. This result aligns completely with the results of current study.

From Figs. 19, 20 and 21, prominent effects of the velocity are marked on the variations of plots of displacement, thermal moment and lateral deflection, respectively. It is evident from Fig. 19 that profiles of displacement field move upward and attain higher values near the field boundary of beam which is clearly the influence of thermal load. Same results have been reported by Şimşek^44^. It is remarked that smaller values of physical fields are obtained for the same numerical value of the velocity of dynamic load in the case of MGT model without fractional derivatives whereas greater values of physical fields are determined in case of MGT model including fractional derivatives. Apart from this, for $\:v=0.05$, highest numerical values of field quantities are achieved and for $\:v=0.03,\:$lowest numerical values of all fields are noticed. Influences of the velocity are prompt in the jumps of the curves of temperature, displacement, thermal moment and lateral deflection for both cases fractional MGT and MGT model of heat transport. These finding underscores the significance of accounting for the relation between velocity and thermal dynamics in the systems at Micro and Nano scale. Earlier, some researchers^45^ investigated that thermal fields are independent from the velocity of travelling thermal load but it is observed from the current investigation that the thermal fields are closely related to the velocity of travelling thermal loading. This is the attractiveness of the present investigation.

## Conclusion

Present research investigates a new, meticulous and novel approach of understanding the thermo-mechanical interactions inside the Nano beams affected from dynamic load with constant velocity as well as ramp type static thermal load in context of Moore Gibson Thompson fractional thermoelastic model including nonlocal elastic effects. The computational analysis of a silicon material based Nano beam has achieved several key findings that can be summarized as follows:

1. MGT and GN-II thermoelastic models reveal smaller values of temperature, displacement, bending moment and lateral deflection fields compared to the classical model (CL model), Lord Shulman thermal model (LS model) and Green and Naghdi-III model (GN-III model). This finding indicates that the smaller values of temperature replicate lower thermal stress inside the system that can support to prevent the structural failure of the system. Besides this, lower stress enhances the sensitivity, capacity and life of the system. Further, it is true that inclusion of fractional derivatives in the thermal conduction model significantly reduces the numerical values of physical fields compared to the conventional heat transport model. Therefore, it is clear that MGT model and GN-II model including fractional derivatives are found to be more efficient to determine the precise and optimum outputs from the devices at Nano scale.
2. Magnitudes of lateral deflection, thermal moment and displacement fields exhibit smaller numerical values for higher numerical value of fractional parameter $\:\alpha\:.$ Lateral deflection is directly proportional to the bending stress. Lower deflection originates the lower values of bending stress and increases the life of the system. Whereas lower values of thermal moment and displacement fields predict high linearity, low energy dissipation, lower stress and higher sensitivity of the sensors.
3. The results stipulate that the greater values of nonlocal parameter depict the stable and smooth behaviour of the physical fields- temperature, displacement, bending moment and lateral deflection. Moreover, greater numerical value of nonlocal parameter suppresses the domain of the field quantities. Consequently, low energy dissipation occurs inside the system that indicates the high sensitivity and better outcomes from the structures and devices.
4. Significant effects of ramp type quantity $\:{t}_{0}$ are perceived on the the curves of all physical fields especially in displacement, thermal moment and deflection. Higher value of ramp type quantity reveals smaller values of the field quantities.
5. In real world applications, several situations are not captured by the static thermal load. And they are defined by travelling thermal load such as Nano-manufacturing, scanning laser heating, moving electric current hotspots in Nano electromechanical resonators, and thermal writing etc. Influences of the velocity of heat input are noticed to be prompt in the jumps of the curves of temperature, displacement, thermal moment and lateral deflection for both cases fractional MGT and MGT model of heat transport. Higher values of velocity of thermal load increases the numerical values of physical fields. This is due to the higher values of kinetic energy.

From the above study, authors believe that this work will provide a new direction to the researchers with profound comprehension of grasping the nonlocal characteristics, static and dynamic thermal loads to modify the functionality and resilience of Nano scale systems so that optimum outcomes can be achieved. By adopting the approach of comparative study of heat conduction theories, size-dependent behaviour and thermal dynamics, this study shows the way to the researchers for the optimum and precise design strategies in NEMS, high accurate engineering assuring better performance, durability and sensitivity in practical applications.

## Data Availability

The datasets used and/or analysed during the current study available from the corresponding author on reasonable request.

## Abbreviations

*λ*, *μ*: Lame constants
*K**: Thermal conductivity rate
*K*: Thermal conductivity
*γ*: Coupling constant
*α*_*t*_: Thermal expansion parameter
*τ*_*q*_: Phase lag for heat flux
*α*: Fractional order quantity
$\theta=T-T_{0}$: Increment in temperature
*T*_0_: Ambient temperature
*δ*: Kronecker’s delta function
*u*_*i*_: Displacement components
*EI*_0_: Flexural rigidity of Euler beam
*e*: Cubical dilatation
*P*: External heat source
$$\:\xi\:$$: Nonlocal parameter
*w*: Lateral deflection
*M*_*T*_: Thermal moment
*v*_*m*_: Thermal diffusivity
*c*_*E*_: Specific heat
*σ*_*ij*_: Nonlocal stress tensor
*h*: Beam thickness
*L*: Length of nanobeam
*b*: Width of beam
*e*_*ij*_: Strain tensor
*ρ*: Density of the material
$\:A=bh$: Area of cross section
$$\:\upsilon\:$$: Poisson ratio
